# Viable healthcare supply chain network design for a pandemic

**DOI:** 10.1007/s10479-022-04934-7

**Published:** 2022-09-09

**Authors:** Mehdi Alizadeh, Mir Saman Pishvaee, Hamed Jahani, Mohammad Mahdi Paydar, Ahmad Makui

**Affiliations:** 1 School of Industrial Engineering, Iran University of Science and Technology, Tehran, Iran; 2 School of Accounting, Business Information, and Supply Chain, RMIT University, Melbourne, Australia; 3 Department of Industrial Engineering, Babol Noshirvani University of Technology, Babol, Iran

**Keywords:** Viable healthcare, Multi-stage stochastic programming, COVID-19, Supply chain network design

## Abstract

The recent COVID-19 pandemic revealed that healthcare networks must have a flexible and effective structure. In this study, we develop a viable healthcare network design for a pandemic using a multi-stage stochastic approach. We propose a multi-level network that includes health centers, computed tomography scan centers, hospitals, and clinics. Patients have conditions to returning to normal life or quarantining at home. Three objectives are defined: maximizing the probability of patient recovery, minimizing the costs of all centers in the network, and minimizing the Coronavirus death rate. We investigate a real case study in Iran to demonstrate the model’s applicability. Finally, we compare the healthcare supply chain network design in a pandemic with a normal situation to advise how the network can continue to remain viable.

## Introduction

In the continuation of human life, the role of healthcare is crucial. From the beginning of humanity, people have died from disease. In all periods, from inception to outbreak, a pandemic disease has been one of the most significant causes of fear and anxiety for the extinction of humankind. In the present era, despite the remarkable progress of technology in healthcare, the prevalence of the Coronavirus has given life to this fear. Indeed, an effective procedure is required for dealing with unknown diseases. The root cause of many pandemic diseases is infection; the virus will move ever more rapidly as long as drugs are not available for the general public.

The Coronavirus outbreak led to the failure of many industries and national economies. It also sent an important message to the world that in the age of technology, there is still not sufficient readiness to cope with communicable diseases—diseases that can directly harm communities. In academic and research streams, more than 80% of the published research about the pandemic supply chain in 2020 indicates the importance of research in this field (Swanson & Santamaria, 2021). The Coronavirus is a serious indication that pandemic diseases can occur at any time and that humans must be prepared ahead of time.

Nowadays in our complex world, decision-making issues are often dynamic, in other words, time and uncertainty play a first role. In this situation, the decision-maker must be able to respond to events with a correct decision policy. Decisions that can respond to realized outcomes that were previously unknown and are modeled under the multi-stage stochastic programming approach (Birge & Louveaux, 2011). Although the two-stage stochastic programming is often adopted in single-period situations, the multi-stage stochastic programming approach to dealing with random data in a dynamic environment (e.g., multi-period) has been introduced. Through multi-stage stochastic programming, data uncertainty is presented through the scenario tree and the objective function is to minimize the total risk imposed on the decision sequence (Zahiri et al., 2017). Researchers recently have shown that multi-stage stochastic planning is a flexible way of describing the problems of optimization under dynamic uncertainty. Various formulations for the multi-stage problem have been proposed in the literature review. In the course of the prevalence of COVID-19, supply chain networks have also undergone a wide range of changes (Tirkolaee et al., 2022). The situation demonstrated that healthcare supply chain networks are in dire need of a crucial flip. An important question that arises is this: How should a healthcare network respond to a severe pandemic event? The network structure is expected to be shaped so that it is still productive in such an event. This study aims to respond to such significant questions as:

- Is the initial normal healthcare supply chain that is already established in a city capable of responding to a pandemic?
- Due to the changes in the severity of the Coronavirus pandemic, how many treatment centers should be activated in a city to care for pandemic patients without causing harm to the treatment process of other patients?
- What is the probability of mortality based on the possibilities and severity of the pandemic, and how can they be reduced?
- How can the likelihood of patient recovery be increased in different medical centers?

This article is organized into seven sections. In the second section (following this introduction), we review the research background along with the research gap, and we consider the contribution of the study. In the third section, the problem is defined, and then the mathematical model is presented according to several constraints and hypotheses. Section 4 focuses on the methodology we used; specifically, a multi-stage stochastic programming approach is developed, and a scenario tree is defined for the problem. Section 5 is dedicated to a real case study, and the input and output data are explained in detail. To show the capability of the proposed model in all possible situations, three numerical problem sets in small, medium, and large dimensions are solved, and the results are discussed. At the end of this section, we perform a sensitivity analysis to review the significant parameters of the model. In the last section, the conclusion is discussed with several future directions for this study.

## Literature review

This study and its proposed model and solution methodology are related to three main streams as follows.

### Healthcare supply chain

This section reviews the most recent articles published regarding the healthcare supply chain. Supply chain network design includes broad literature (Gholizadeh et al. 2021; Homayouni et al. 2021), in which the healthcare network is of utmost importance. Many articles exist in this domain (Mathur et al. 2018); however, the idea of disruption in the chain is rarely discussed. Aldrighetti et al. (2019) propose an approach to analyzing the effects of disruption on the healthcare system. They test different strategies, taking into account the highest level of service and the prevention of shortages in hospitals. They believe that having a backup supplier is crucial in the event of a long-term disruption. Scavarda et al. (2019) analyze the healthcare supply chain network in developing countries, emphasizing the social responsibility of health centers. Alizadeh et al., (2020a, 2020b) design the direct and reverse distribution of medical consumer supplies by presenting the bounded De Novo programming approach to the issue. They approach the issue of healthcare from another angle, considering the biological risk. Another vital part of a healthcare supply chain is the drug supply chain. In this vein, Sazvar et al. (2021) present a model for a closed-loop drug supply chain network in a pandemic. They consider that expired medicines should be buried, reprocessed, or recycled. The authors only consider demand uncertainty and no other disruption event is formulated in their model.

### Pandemic effects on supply chains

Pandemics are always a possibility, even if they have not occurred for a long time. Therefore, policies have to be pre-prepared so that the supply chain network can work without disruption when the pandemic transpires. The availability of jobs in a pandemic sometimes disrupts the supply chain because people quit their jobs for fear of spreading the disease (Nagurney, 2021). The COVID-19 pandemic has put a great deal of pressure on supply chains—one of the most severe consequences of which is a decline in labor resources (Paul, & Chowdhury, 2020). In addition, the production capacity of these companies is reduced due to political decisions such as reducing office hours and halting the hiring of employees in order to maintain social distance in the office (Leite & Kumar, 2020). Therefore, to maintain social distancing and reduce the risk of disease, employees cannot work full time, thus reducing the active workforce (Trautrims et al., 2020). As labor is an essential input to any economic activity of the supply chain network, its reduction can lead to increased costs, reduced profits, and unrealized demand (Jaillet et al 2018).

Limited factory operations can also cause productive equipment failure and loss of capital (Dente & Hashimoto, 2020). The healthcare supply chain suffers from all these effects, as well. However, an effective network design will lessen the impact. Noting that the impact on global healthcare systems is profound, Iyengar et al. (2020) believe that the major challenges to managing a public health crisis are the production and distribution of medical equipment, surgical equipment, and medicines for people on the clinical front lines. Expressing a new concept called agility, Ivanov (2020) notes that variability, flexibility, and sustainability could help companies recover and rebuild supply chains after long-term pandemic diseases such as COVID-19. Ivanov and Das (2020) describe the speed of the pandemic’s spread, the duration of production, distribution, and disruption of the market, and the decline in demand due to the effects of the Coronavirus on the global supply chain. With this in mind, they presented plans to improve the global supply chain. Nagurney et al. (2021) also note that the global pandemic has affected economic and social activities worldwide and that the implications for healthcare workers and patients are critical. Khalilpourazari and Hashemi Doulabi (2021a, 2021b) showed that increasing the capacity of the test increased the diagnosis of asymptomatic cases and limited the transmission of the disease to the community. In addition, they performed many sensitivity analyses to discover the effects of changes in transmission rates on epidemic growth.

Pandemics can lead to fierce global competition for medical supplies. Sousa Jabbour (2020) considers recovery and learning as endeavors that should be prioritized by companies under the pressure of COVID-19. In transportation management, trips have been disrupted due to health protocols such as traffic restrictions during the pandemic. In the agricultural industry, in particular, this can be a significant setback (Gray, 2020). Delay in transferring goods in the supply chain disrupts the global trade supply chain (Deaton et al., 2020), and any delay may have serious consequences in even a short time (Chiaramonti et al., 2020).

The effects of the epidemic on the supply chain have also been investigated in pre-Covid-19 articles (Dasaklis et al., 2012), and studies in this area have increased significantly with the prevalence of the Coronavirus and its widespread effects. To respond to industry requirements, researchers have adapted their research to new pandemic statuses [e.g., Chowdhury et al. (2021), Khalilpourazari and Doulabi (2021), Nikolopoulos et al. (2021), Khalilpourazari et al. (2021), Alizadeh et al. (2021), and Armani et al. (2020)].

### Modeling supply chains in a pandemic

Currie et al. (2020) examine new challenges in line with the pandemic COVID-19 to show the effectiveness of similar models in decision-making. Craighead et al. (2020) examine how organizations respond to the epidemic and how supply chains and related processes should be adjusted in the event of another pandemic. They present a game theory model to compete in preparing medical goods in a pandemic. Shahed et al. (2021) present a multi-stage mathematical model for reducing disruption in natural disasters, including the COVID-19 pandemic. Their purpose is to manage the supply chain in a global situation where disruption can harm both the seller and the retailers. Li et al. (2021) present a mathematical model to investigate the effects between infectious disease dynamics and supply chain disruption. After analyzing the supply chain network, they show that time-sensitive containment strategies can prevent pandemic and economic damage as much as possible. They believe that a lean resource allocation strategy can significantly reduce the impact of supply chain shortages. The pandemic supply chain network design differs from natural disaster cases like earthquakes. Salama & McGarvey (2021) study supply chains for critical social products separately to examine the impact of supply chain network expansion on maximizing the met demand. They present a linear programming model of stochastic mixed integers to maximize the CVar. Finally, they investigate the effects of diversifying network node locations in different office areas on supply chain performance. Policymakers can use the capacity of the health care system and resource allocation to try to minimize optimal mortality. Khalilpourazari and Hashemi Doulabi (2021a, b) in their research design a new hybrid reinforcement learning-based algorithm capable of solving complex optimization problems. They apply their algorithm to several criteria and show that the proposed method provides quality solutions for the most complex criteria. Poursoltan et al. (2021) propose a green closed-loop supply chain framework for ventilators for the COVID-19 pandemic. Their proposed model, which is based on a case study, stimulates ventilator production. The model includes environmental stability to limit carbon emissions as a constraint. A new stochastic optimization model is explained with strategic and tactical decision-making for this closed-loop supply chain network design problem. Lotfi et al. (2022) concede that in a high disruption and uncertain situation like Corona pandemic, the best strategy for reducing inventory costs could be the vendor-managed inventory (VMI) policy. They propose a robust fuzzy, data-driven optimization for the supply chain and consider sustainable and resilience network for their proposed health care system. They believe that their VMI system can efficiently deal with uncertainty and disruption.

### Contributions of this study

The above investigation of the literature reveals that many articles have been published in the field of the healthcare supply chain, but that studies considering disruption are rare. Chowdhury et al. (2021) confirm that considering a pandemic is much different from disruption in supply chain models and needs a dedicated assumption. Pandemics occur suddenly and infrequently, and it is necessary to consider their unique effects on the chain. During the COVID-19 pandemic, several articles have been published considering its consequences on supply chains (e.g. Govindan et al., 2020; Nikzamir & Baradaran, 2020). However, we found no research that designs a viable healthcare supply chain network that effectively works in both pandemic and normal statuses.

In this research, we attempt to provide a viable healthcare supply chain network design using a mathematical model that is responsive even in a pandemic. Future events occur under different scenarios, each determining its own circumstances; therefore, each requires unique planning. Additionally, a center’s capabilities are not always constant and may change depending upon the scenario. In this study, a dynamic capacity allocation strategy is employed using a multi-stage stochastic programming model. In every healthcare supply chain, hospitals are susceptible centers; a few minor changes can bring life back to a person or take it away. For this reason, it is necessary to pay close attention to variances in hospital capacity. A dynamic capacity means that the center’s capacity is updated over time, and for a hospital, this means making a note of who entered the hospital, who left the hospital, and who remained in the hospital in each period. Our investigation also revealed no articles that study dynamic capacity in multi-stage stochastic programming for a healthcare supply chain.

In this study, we also consider a risk level (related to the number of people with COVID-19) for each geographical area (e.g., a province of a country) in which the model is performed. In the solution process, different conditions are examined to give a comprehensive view of the existing risk level (status) in the province.

## Problem formulation

### Problem description

The high risk of Coronavirus disease causes a high level of stress for people, which pushes them to go to health centers at the earliest opportunity and check the status of the disease using detection kits or lung scans. Of course, people generally receive a CT scan after seeing a doctor. For this purpose, there is a medical center at the beginning of the supply chain network. In the current situation, suspected cases go to health centers. If their polymerase chain reaction (PCR) test is positive, the doctor will refer them to a CT scan center or to a hospital that has lung scan machines for the detection of complications. If the patient’s lungs are in danger, the patient is referred to the hospital. If the test at the health center is negative, the case is considered a healthy person and is removed from the network.

If the patient is transferred from a health center to a CT scan center after a positive test, the doctor will decide whether the person should be hospitalized or quarantined at home based on the extent of the lung concerns. Of course, some patients will go directly to CT scan centers, and there will be heavy traffic in some centers for that reason. This can lead to disruption in the chain. Therefore, people will be removed from the chain after a CT scan unless a problem is detected in their lungs.

**Fig. 1:**
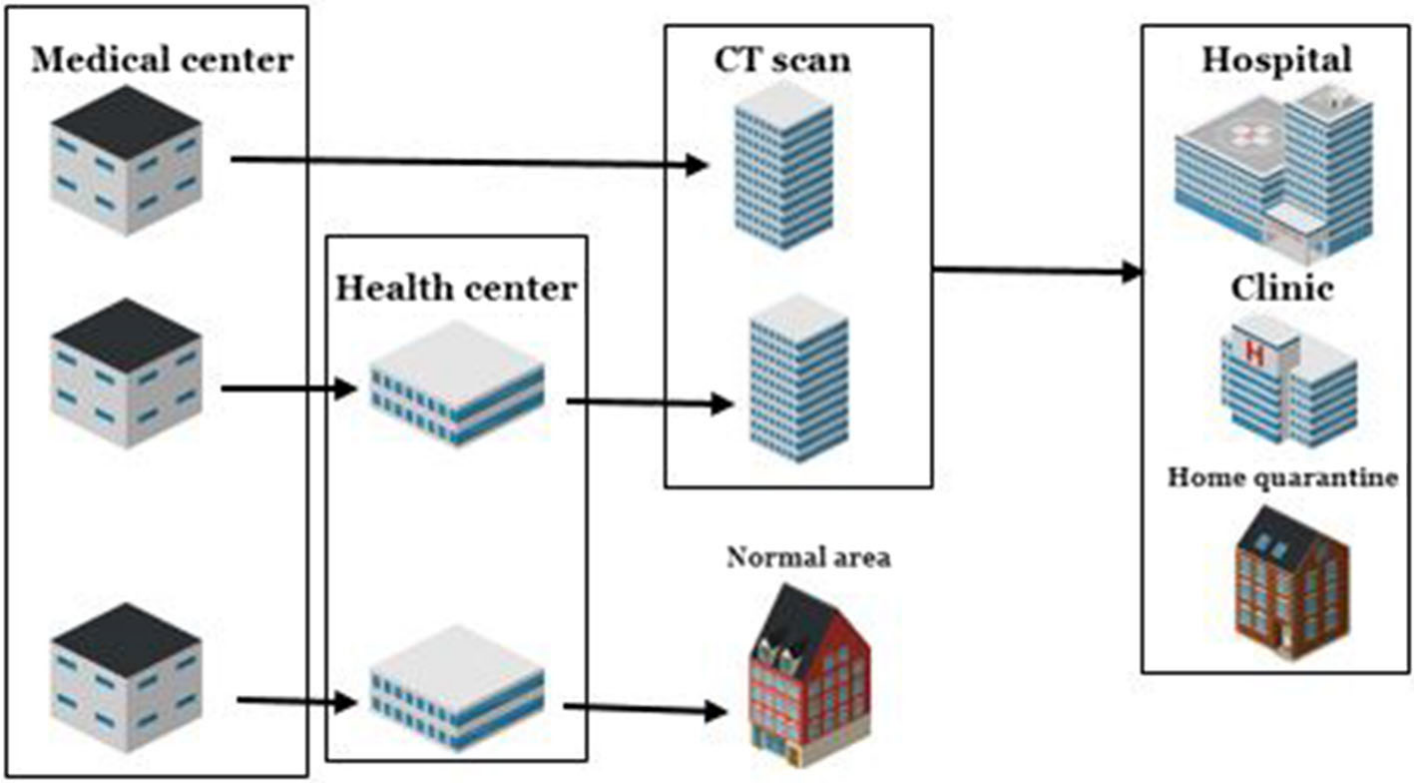
A schematic view of a healthcare supply chain network design during the pandemic

Hospitals also have CT scan centers that are publicly run, and they cost patients less than clinics. On the other hand, clinics tend to have better spaces, and patients prefer them if they can afford the expense. After a certain period of time, the patient undergoes a Coronavirus test in the same center according to his/her doctor’s diagnosis for a follow-up. If their PCR test is still positive, but the patient’s general status is acceptable, the patient will go to home quarantine. If the test is negative, the patient will leave the chain a healthy person. A schematic view of the problem is shown in Fig. 1.

As mentioned before, the restrictions applied in each city vary depending on time; if the number of infected people increases over a period of time, health centers and then CT scan centers and hospitals will be disrupted. All hospitals are prepared for critical conditions and hospitalization of patients, but to improve the healthcare system’s performance for all patients, only some hospitals are considered to serve Coronavirus patients. If the capacity of a hospital cannot meet the demand of patients, the nearest hospital will be activated according to the capacity and priority of the prior hospital. CT scan centers are in the same situation, and additional CT scan centers will be activated for patients if necessary. The capacity of a health center and a CT scan center are determined according to the maximum number of people that can be visited during their working time. The capacity of hospitals and clinics is determined based on the number of beds available for allocation to Coronavirus patients.

In every country or city, various risk levels (statuses) are considered based on the number of active cases at any period. The levels are illustrated with colors (blue, yellow, orange, and red). Under these circumstances, different scenarios can directly affect the chain mechanism. If the number of infected people increases exponentially in a certain period of time, the status worsens (perhaps to red). If the status returns to normal, the disruption issue will still exist because many people in quarantine may not follow health protocols.

This study aims to provide a network of healthcare supply chains in pandemic risk levels to simultaneously increase the probability of patient recovery, reduce costs, and minimize the probability of the risk of under-capacity mortality. When a patient is tested positive, he or she wants to survive and recover as quickly as possible. Therefore, according to the performance of the hospitals in the region and, of course, the amount of occupied capacity, that hospital will decide. Costs play an important role but are not a top priority. The patient does not necessarily go to the nearest hospital, but to a hospital that has a better chance of recovery.

### Mathematical model

The following notations are used to formulate the above-mentioned healthcare supply chain. We also note the unit of each parameter at the end of its definition in parentheses. It is worthy to mention that the monetary units of costs are in million Iranian Rial (MIRR). The percentages can be estimated by using COVID-19 historical data with a data analytics prediction model like classification, clustering, or time series (c.f. Jahani et al., 2022).

*Sets and indices*

$$c$$
Candidate CT scan center (c = 1,2,..,C).
$$k$$
Candidate health center (k = 1,2,..,K).
$$h$$
Candidate hospital (h = 1,2,..,H).
$$j$$
Candidate clinic (j = 1,2,..,J).
$$w$$
Home quarantine area (w = 1,2,..,W).
$$r$$
Normal area (r = 1,2,..,R
$$t$$
Time period (t = 1,2,..,T).

*Parameters*

$$ck_{kt}$$
Cost of activating health center $k$ in period $t$ (MIRR).
$$cc_{ct}$$
Cost of activating CT scan center $c$ in period $t$ (MIRR).
$$ch_{ht}$$
Cost of activating hospital $h$ in period $t$ (MIRR).
$$cj_{jt}$$
Cost of activating clinic $j$ in period $t$ (MIRR).
$$capk_{k}$$
Capacity of health center $k$(Number of available beds).
$$capc_{c}$$
Capacity of CT scan $c$(Number of referrals to CT scan).
$$pk_{kt}$$
Probability of infection in health center $k$ in period $t$ (%).
$$pc_{ct}$$
Probability of infection in CT scan center $c$ in period $t$ (%).
$$ph_{ht}$$
Probability of improvement in hospital $h$ in period $t$ (%).
$$pj_{jt}$$
Probability of improvement in clinic $j$ in period $t$ (%).
$$u$$
Probability of death (mortality risk) of COVID-19 (%).
$$yj_{jt}$$
Probability of death in clinic $j$ based on the capacity used in period $t$ (%).
$$yh_{ht}$$
Probability of death in hospital $h$ based on the capacity used in period $t$ (%).
$$d_{t}$$
Number of patients in period $t$ (#).
$$\alpha_{t}$$
Percentage of patients who go to a health center in period $t$ (%).
$$\beta_{t}$$
Percentage of patients who go from a health center to the hospital in period $t$ (%).
$$\varepsilon_{t}$$
Percentage of community that has not been infected with the disease in period $t$ (%).
$$\varphi_{t}$$
Percentage of patients who go to a CT scan center after a health center in period $t$ (%).
$$\lambda_{t}$$
Percentage of patients who go to a hospital after the CT scan in period $t$ (%).
$$\gamma_{t}$$
Percentage of patients who go to a clinic after the CT scan in period $t$ (%).
$$\mu_{t}$$
Percentage of patients who go to a home quarantine after a CT scan in period $t$ (%).

*Decision variables*

$$xk_{kt}$$
1, if the health center $k$ is activated in period $t$; otherwise, 0.
$$xc_{ct}$$
1, if the CT scan center $c$ is activated in period $t$; otherwise, 0.
$$xh_{ht}$$
1, if the hospital $h$ is activated in period $t$; otherwise, 0.
$$xj_{jt}$$
1, if the clinic $j$ is activated in period $t$; otherwise, 0.
$$qik_{kt}$$
Number of patients visiting the health center $k$ in period $t$.
$$qic_{ct}$$
Number of patients visiting the CT scan center $c$ in period $t$.
$$qkc_{kct}$$
Number of patients go from the health center $k$ to the CT scan center $c$ in period $t$.
$$rkh_{kht}$$
Number of patients go from the health center $k$ to the hospital $h$ in period $t$.
$$rkr_{krt}$$
Number of patients in the health center $k$ with negative PCR test results and return to normal area $r$ in period $t$.
$$sch_{cht}$$
Number of patients go from the CT scan center $c$ to hospital $h$ in period $t$.
$$scj_{cjt}$$
Number of patients go from the CT scan center $c$ to clinic $j$ in period $t$.
$$scw_{cwt}$$
Number of patients go from the CT scan center $c$ to home quarantine $w$ in period $t$.
$$caph_{ht}$$
Capacity of the hospital $h$ in period $t$.
$$capj_{jt}$$
Capacity of the clinic $j$ in period $t$.

It is worth noting that the probability of death in a clinic or a hospital due to its used capacity is calculated by $u*(\frac{{capj_{t} }}{{capj_{first} }})$ and $u*(\frac{{caph_{t} }}{{caph_{first} }})$, respectively.

#### Objective functions

1 $$ MAXz_{1} = \sum\limits_{h} {\sum\limits_{t} {ph_{ht} } } xh_{ht} + \sum\limits_{j} {\sum\limits_{t} {pj_{jt} } } xj_{jt} - \left( {\sum\limits_{k} {\sum\limits_{t} {pk_{kt} } } xk_{kt} + \sum\limits_{c} {\sum\limits_{t} {pc_{ct} } } xc_{ct} } \right) $$

2 $$ MINz_{2} = \sum\limits_{k} {\sum\limits_{t} {ck_{kt} } } xk_{kt} + \sum\limits_{c} {\sum\limits_{t} {cc_{ct} } } xc_{ct} + \sum\limits_{h} {\sum\limits_{t} {ch_{ht} } } xh_{ht} + \sum\limits_{j} {\sum\limits_{t} {cj_{jt} } } xj_{jt} $$

3 $$ MINz_{3} = \sum\limits_{h} {\sum\limits_{t} {yh_{ht} } } xh_{ht} + \sum\limits_{j} {\sum\limits_{t} {yj_{jt} } } xj_{jt} $$

Equation (1) aims to minimize the prevalence of the disease in health centers and CT scan centers. This will happen by maximizing the improvement ratios and minimizing the infection ratios at hospitals and clinics depending on their conditions and facilities. Equation (2) minimizes the total cost of activation of centers. In reality, many health centers exist in a healthcare chain. However, to prevent the spread of Coronavirus, it is required to enable a minimum number of healthcare centers for COVID-19 patients. Equation (3) minimizes the rate of the probability mortality in all hospitals and clinics. The failure rates in defeating the virus (death rate) in centers are directly related to the facilities and capacities of the selected centers. When the capacity of one center is completely full, it can increase the probability of mortality for two reasons: first, the patient's time to reach the next center is longer, and second, more clients will use more facilities. This will result in congestion in facilities and increase the probability of mortality.

#### Constraints

4 $$ d_{t} = \sum\limits_{k} {\alpha_{t} } qik_{kt} + \sum\limits_{c} {(1 - } \alpha_{t} )qic_{ct} \;\;\forall t $$

5 $$ \beta_{t} qik_{kt} = \sum\limits_{h} {rkh_{kht} } \;\;\forall t,k $$

6 $$ \varepsilon_{t} qik_{kt} = \sum\limits_{r} {rkr_{krt} } \;\;\forall t,k $$

7 $$ \varphi_{t} qik_{kt} = \sum\limits_{c} {qkc_{kct} } \;\;\forall t,k $$

8 $$ \lambda_{t} \left( {(\sum\limits_{k} {qkc_{kct} ) + qic_{ct} } } \right) = \sum\limits_{h} {sch_{cht} } \;\;\forall t,c $$

9 $$ \gamma_{t} \left( {(\sum\limits_{k} {qkc_{kct} ) + qic_{ct} } } \right) = \sum\limits_{j} {scj_{cjt} } \;\;\forall t,c $$

10 $$ \mu_{t} \left( {(\sum\limits_{k} {qkc_{kct} ) + qic_{ct} } } \right) = \sum\limits_{w} {scw_{cwt} } \;\;\forall t,c $$

11 $$ qik_{kt} \le capk_{k} x_{kt} \;\;\forall t,k $$

12 $$ qic_{ct} + \sum\limits_{k} {qkc_{kct} } \le capc_{c} xc_{ct} \;\;\forall t,c $$

13 $$ \sum\limits_{k} {rkh_{kht} } + \sum\limits_{c} {sch_{cht} } \le caph_{ht} xh_{ht} \;\;\forall t,h $$

14 $$ caph_{ht} = caph_{h(t - 1)} + \left( {\sum\limits_{k} {rkh_{kh(t - 2)} } + \sum\limits_{c} {sch_{ch(t - 2)} } } \right) - \left( {\sum\limits_{k} {rkh_{kh(t - 1)} } + \sum\limits_{c} {sch_{ch(t - 1)} } } \right)\;\;\forall t,h $$

15 $$ \sum\limits_{c} {scj_{cjt} } \le capj_{jt} xj_{jt} \;\;\forall t,j $$

16 $$ capj_{jt} = capj_{j(t - 1)} + \left( {\sum\limits_{c} {scj_{cj(t - 2)} } } \right) - \left( {\sum\limits_{c} {scj_{cj(t - 1)} } } \right)\;\;\forall t,j $$

Constraint (4) calculates the total number of patients who visit either health centers or CT scan centers. Constraints (5) to (7) are a set of equilibrium constraints for health centers’ input and output flow. These restrictions state that the number of people who exit from a health center is calculated by the people who enter other related centers (CT scans or hospitals) or return to normal life in the community. Constraints (8) to (10) are also in equilibrium. In other words, the number of patients in a hospital is equal to the number of patients who enter the hospital from its related health centers and CT scans. There is also a balance for clinics and quarantine. Constraints (11) and (12) indicate the capacity of health centers and CT scan centers. Constraint (13) indicates the capacity of hospitals. Constraint (14) calculates the capacity of a hospital in each period by the remaining capacity from the previous period, the number discharged two periods ahead, and the number of patients admitted in the prior period. Constraint (15) indicates the capacity of a clinic, and Constraint (16) calculates the capacity of a clinic in each period by the remaining capacity from the previous period, the number of patients discharged two periods ahead, and the number of patients admitted in the prior period. It is also clear for the defined percentages that $\beta_{t} + \varepsilon_{t} + \varphi_{t} = 1$ and $\lambda_{t} + \gamma_{t} + \mu_{t} = 1$.

The capacity of hospitals and clinics changes dynamically over time. These centers initially have a basic capacity, but in the following periods, this will change according to patient input and output flows. Therefore, the capacity of centers at each stage should be updated according to the new conditions. We use a multi-stage optimization model with the dynamic capacity to update the capacities in each stage according to the previous period and the current scenario.

## Solution methodology

In reality, various events can change a plan over time, and scenarios can be imagined in the case of uncertainties. In this study, the problem is managing the number of patients with Coronavirus symptoms. This number could increase or decrease in a day; this is subject to compliance with individual health protocols. Different scenarios occur during each time period (stage) and reflect the problem as multi-stage stochastic programming. In the past, stochastic programming approaches were only available in two periods (Kall et al., 1994) and were still employed in stochastic modeling (Jahani et al., 2021). In the health field, especially when a pandemic occurs, it is vital to know the scenarios of future periods. Multi-stage stochastic programming is superior to two-stage stochastic programming because the pandemic risks are mitigated over several periods. In the proposed problem of this study, the level of risk in each region causes uncertainty, and the statuses may remain constant or change completely, from a good status (blue color) to a bad status (red color). Therefore, our solution methodology will cover such uncertain situations.

### Multi-stage stochastic programming

Two-stage stochastic programming can be extended to multi-stage stochastic programming for a multi-period optimization model (Kall et al., 1994). Uncertainty in these approaches is represented by a scenario tree (Kashanian et al., 2020). At the beginning of the decision-making period $t$, decision $x_{0}$ is made. In multi-stage stochastic programming, all levels are considered at the same time. At this stage, the investment and operation costs of all levels may not be ideal, but the overall cost is optimal.

**Fig. 2:**
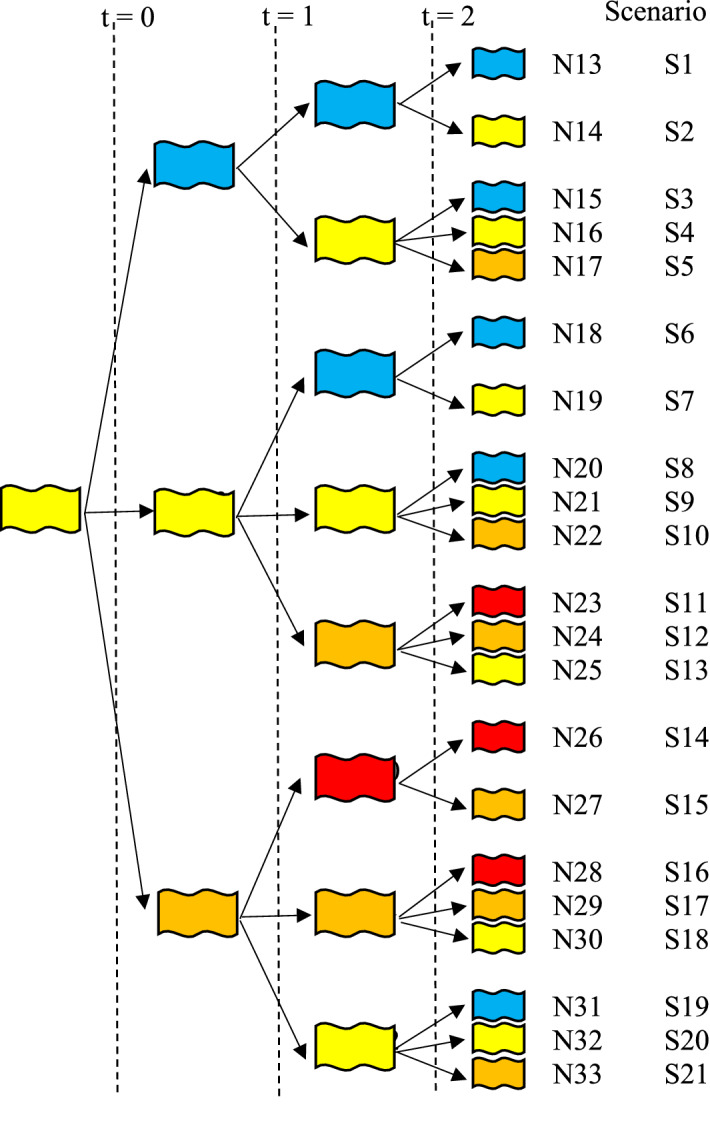
Scenario tree used for the proposed multi-stage programming

The scenario tree for the proposed multi-stage stochastic programming model is shown in Fig. 2. We consider four stages equivalent to four periods. Each node represents one of the risk statuses (blue, yellow, orange) that occur in a pandemic. In the first node, the status is yellow, and in the next period, the status either gets better (turns blue), does not change (remains yellow), or gets worse (turns orange). This is repeated at each stage. In other words, the evolution of the stochastic process of the scenario tree from root to leaf node is carried out in the same way, and each of them is called a scenario. According to Fig. 2, the proposed problem contains 21 scenarios. It is worthwhile to mention that the risk levels are defined at the country level. In our case study, based on information from the Iran Ministry of Health and Medical Education, we defined the colors according to the number of people suspected of being infected with the Coronavirus. These colors are written in order of good to bad risk statuses. Under these statuses, a sudden color change is not expected during each time period. In other words, after each period, the color will change step by step. For instance, if the situation in the current period is orange, the next period will either be worse (red), better (yellow), or without change (orange).

For our multi-stage model, the decision variables and some parameters affected by the risk levels are indexed over each scenario. The following notations are used to formulate the multi-stage stochastic model considering uncertainty.

#### New indices

$s$ Index of scenarios.

*New parameters*

$$pk_{kts}$$
Probability of infection in the health center $k$ in the period of $t$ under the scenario $s$.
$$pc_{cts}$$
Probability of infection in the CT scan center $c$ in the period of $t$ under the scenario $s$.
$$ph_{hts}$$
Probability of improvement in the hospital $h$ in the period of $t$ under the scenario $s$.
$$pj_{jts}$$
Probability of improvement in the clinic $j$ in the period of $t$ under the scenario $s$.
$$yj_{jts}$$
Probability of death in the clinic $j$ based on the capacity used in the period of $t$ under the scenario $s$; = $u*(\frac{{capj_{t} }}{{capj_{first} }})$.
$$yh_{hts}$$
Probability of death in the clinic $j$ based on the capacity used in the period of $t$ under the scenario $s$; = $u*(\frac{{capj_{t} }}{{capj_{first} }})$.
$$d_{ts}$$
Number of patients in the period of $t$ under the scenario $s$.
$$\alpha_{st}$$
Percentage of patients who go to the health center in the period of $t$ under the scenario $s$.
$$\beta_{st}$$
Percentage of patients who go from the health center to the hospital in the period of $t$ under the scenario $s$.
$$\varepsilon_{st}$$
Percentage of patients not infected with the disease in the period of $t$ under the scenario $s$.
$$\varphi_{st}$$
Percentage of patients who go to the CT scan center after the health center in the period of $t$ under the scenario $s$.
$$\lambda_{st}$$
Percentage of patients who go to the hospital after the CT scan center in the period of $t$ under the scenario $s$.
$$\gamma_{st}$$
Percentage of patients who go to the clinic after the CT scan center in the period of $t$ under the scenario $s$.
$$\mu_{st}$$
Percentage of patients who go to home quarantine after the CT scan center in the period of $t$ under the scenario $s$.

*New decision variables*

$$qik_{kts}$$
Number of patients visiting health center $k$ in the period of $t$ under the scenario $s$.
$$qic_{cts}$$
Number of patients visiting the CT scan center $c$ in the period of $t$ under the scenario $s$.
$$qkc_{kcts}$$
Number of patients who go from the health center $k$ to the CT scan center $c$ in the period of $t$ under the scenario $s$.
$$rkh_{khts}$$
Number of patients who go from the health center $k$ to the hospital $h$ in the period of $t$ under the scenario $s$.
$$rkr_{krts}$$
The number of patients in the health center $k$ who have negative test results and then return to normal life $r$ in the period of $t$ under the scenario $s$.
$$sch_{chts}$$
Number of patients who go from the CT scan center $c$ to the hospital $h$ in the period of $t$ under the scenario $s$.
$$scj_{cjts}$$
Number of patients who go from the CT scan center $c$ to the clinic $j$ in the period of $t$ under the scenario $s$.
$$scw_{cwts}$$
Number of patients who go from the CT scan center $c$ to home quarantine $w$ in the period of $t$ under the scenario $s$.

#### New objective functions

17 $$ MAXf_{1} = \sum\limits_{s} {\pi_{s} } \left( {\sum\limits_{h} {\sum\limits_{t} {ph_{hts} } } xh_{ht} + \sum\limits_{j} {\sum\limits_{t} {pj_{jts} } } xj_{jt} - \left( {\sum\limits_{k} {\sum\limits_{t} {pk_{kts} } } xk_{kt} + \sum\limits_{c} {\sum\limits_{t} {pc_{cts} } } xc_{ct} } \right)} \right) $$

18 $$ MINf_{2} = \sum\limits_{s} {\pi_{s} } \left( {\sum\limits_{k} {\sum\limits_{t} {ck_{kt} } } xk_{kt} + \sum\limits_{c} {\sum\limits_{t} {cc_{ct} } } xc_{ct} + \sum\limits_{h} {\sum\limits_{t} {ch_{ht} } } xh_{ht} + \sum\limits_{j} {\sum\limits_{t} {cj_{jt} } } xj_{jt} } \right) $$

19 $$ MINf_{3} = \sum\limits_{s} {\pi_{s} } \left( {\sum\limits_{h} {\sum\limits_{t} {yh_{hts} } } xh_{ht} + \sum\limits_{j} {\sum\limits_{t} {yj_{jts} } } xj_{jt} } \right) $$

#### New constraints

20 $$ d_{ts} = \sum\limits_{k} {\alpha_{st} } qik_{kts} + \sum\limits_{c} {(1 - } \alpha_{st} )qic_{cts} \;\;\forall s,t $$

21 $$ \beta_{st} qik_{kts} = \sum\limits_{h} {rkh_{khts} } \;\;\forall s,t,k $$

22 $$ \varepsilon_{st} qik_{kts} = \sum\limits_{r} {rkr_{krts} } \;\;\forall s,t,k $$

23 $$ \varphi_{st} qik_{kts} = \sum\limits_{c} {qkc_{kcts} } \;\;\forall s,t,k $$

24 $$ \lambda_{st} \left( {(\sum\limits_{k} {qkc_{kcts} ) + qic_{cts} } } \right) = \sum\limits_{h} {sch_{chts} } \;\;\forall s,t,c $$

25 $$ \gamma_{st} \left( {(\sum\limits_{k} {qkc_{kct} ) + qic_{ct} } } \right) = \sum\limits_{j} {scj_{cjts} } \;\;\forall s,t,c $$

26 $$ \mu_{st} \left( {(\sum\limits_{k} {qkc_{kct} ) + qic_{ct} } } \right) = \sum\limits_{w} {scw_{cwts} } \;\;\forall s,t,c $$

27 $$ qik_{kt} \le capk_{k} x_{kt} \;\;\forall s,t,k $$

28 $$ qic_{cts} + \sum\limits_{k} {qkc_{kcts} } \le capc_{c} xc_{ct} \;\;\forall s,t,c $$

29 $$ \sum\limits_{k} {rkh_{khts} } + \sum\limits_{c} {sch_{chts} } \le caph_{ht} xh_{ht} \;\;\forall s,t,c $$

30 $$ caph_{ht} = caph_{h(t - 1)} + \left( {\sum\limits_{k} {rkh_{kh(t - 2)s} } + \sum\limits_{c} {sch_{ch(t - 2)s} } } \right) - \left( {\sum\limits_{k} {rkh_{kh(t - 1)s} } + \sum\limits_{c} {sch_{ch(t - 1)s} } } \right)\;\;\forall s,t,h $$

31 $$ \sum\limits_{c} {scj_{cjts} } \le capj_{jt} xj_{jt} \;\;\forall s,t,j $$

32 $$ capj_{jt} = capj_{j(t - 1)} + \left( {\sum\limits_{c} {scj_{cj(t - 2)s} } } \right) - \left( {\sum\limits_{c} {scj_{cj(t - 1)s} } } \right)\;\;\forall s,t,j $$

### LP metric method

The LP method is one of the classic methods for solving multi-objective problems. This method is still under consideration for two reasons: First, this method requires very little information from the decision and does not depend on the decision-maker. Second, it is easily understandable and useful for practical models (Alizadeh et al., 2020a, b). We found the LP-metrics method as one of the most used approaches, which is also more perceptible for executives compared to other methods, and in many cases of using this method such as Shabbir et al. (2021), Alinezhad et al. (2022) and Jabarzadeh et al. (2020), acceptable results have been provided.

This method tries to minimize the bias of objective functions relative to an ideal solution. It provides the best possible answer that results in the smallest distance from an ideal point. An ideal answer achieves the optimal value of all objectives simultaneously. The optimum point is represented as:

33 $$ F(x^{*} ) = \left\{ {f_{1} (x^{*} ),f_{2} (x^{*} ),...,f_{n} (x^{*} )} \right\} $$

That $F(x^{*} )$ shows the optimal performance of all targets. So that $x^{*}$ can optimize each $f_{1} (x^{*} )$. In practice, there is no $x^{*}$ due to conflict between the objectives of the problem. In the same way, the metric distance is used by measuring the proximity of an available solution to the ideal solution. This criterion is defined as a compatible function as follows. For “the less, the better” issues, the compatible function is defined as:

34 $$ L_{p} = \left\{ {\sum\limits_{i = 1}^{n} {W_{i} } \left( {f_{i} (x_{i} )} \right) - \left( {f_{i} (x_{i}^{\min } )} \right)^{p} } \right\}^{\frac{1}{p}} $$

For the problem of “the more, the better,” the compatible function is defined as:

35 $$ L_{p} = \left\{ {\sum\limits_{i = 1}^{n} {W_{i} } \left( {f_{i} (x_{i}^{\max } )} \right) - \left( {f_{i} (x_{i} )} \right)^{p} } \right\}^{\frac{1}{p}} $$

$x_{i}^{\max }$ and $x_{i}^{\min }$ represent the ideal solution in the optimization of the *i*_*th*_ objective and $x_{i}$ represents the assumed solution. $W_{i}$ represents the degree of importance for the purpose of *i*_*th*_ ($W_{i} \ge 0$). The compatible function, $L_{p}$, must be minimized in order to lower the deviations from the ideal solution. The higher the $p$ value, the greater the emphasis on the largest deviations. The $p$ value depends on the decision-maker’s subjectivity. Usually, values of $p =$ 1, $p =$ 2, and $p = \infty$ are used in the formula. The compatible function $L_{p}$ can be related to different objectives with different scales. For the “the less, the better” issues, the compatible function is defined as:

36 $$ L_{p} = \left\{ {\sum\limits_{i = 1}^{n} {W_{i} } \left( {\frac{{\left( {f_{i} (x_{i} )} \right) - \left( {f_{i} (x_{i}^{\min } )} \right)}}{{\left( {f_{i} (x_{i}^{\min } )} \right)}}} \right)^{p} } \right\}^{\frac{1}{p}} $$

For the problem of “the more, the better,” the compatible function is defined as:

37 $$ L_{p} = \left\{ {\sum\limits_{i = 1}^{n} {W_{i} } \left( {\frac{{\left( {f_{i} (x_{i}^{\max } )} \right) - \left( {f_{i} (x_{i} )} \right)}}{{\left( {f_{i} (x_{i}^{\max } )} \right)}}} \right)^{p} } \right\}^{\frac{1}{p}} $$

The concept of the ideal solution was proposed in 1982 by Zeleny (1976). The anti-ideal solution of the objective functions is shown as follows:

38 $$ F(\overline{x}^{*} ) = f_{1} (\overline{x}^{*} ),f_{2} (\overline{x}^{*} ),...,f_{n} (\overline{x}^{*} ) $$

Based on this concept, the compatible function (distance metric) is applicable in two situations. For “the less, the better” issues, the compatible function is defined as follows:

39 $$ L_{p} = \left\{ {\sum\limits_{i = 1}^{n} {W_{i} } \left( {\frac{{\left( {f_{i} (x_{i} )} \right) - \left( {f_{i} (x_{i}^{\min } )} \right)}}{{\left( {f_{i} (\overline{x}_{i}^{\max } )} \right) - \left( {f_{i} (x_{i}^{\min } )} \right)}}} \right)^{p} } \right\}^{\frac{1}{p}} $$

For the problems of “the more, the better,” the compatible function is determined as follows:

40 $$ L_{p} = \left\{ {\sum\limits_{i = 1}^{n} {W_{i} } \left( {\frac{{\left( {f_{i} (x_{i}^{\max } )} \right) - \left( {f_{i} (x_{i} )} \right)}}{{\left( {f_{i} (x_{i}^{\max } )} \right) - \left( {f_{i} (\overline{x}_{i}^{\min } )} \right)}}} \right)^{p} } \right\}^{\frac{1}{p}} $$

## Model implementation

### Case study

Pandemics always inflict maximum damage on tourist cities because people travel there without knowing they are infected with the disease, and this may cause an outbreak. Mazandaran is one of the main tourist destinations in Iran. Mazandaran has a beautiful landscape; hence, people use the shortest possible travel time to vacation there. Over the COVID-19 pandemic, Mazandaran province has often been in red status, and this has caused irreparable trouble for the population of this region. Mazandaran is one of Iran’s provinces that has most deeply felt these effects.

**Fig. 3:**
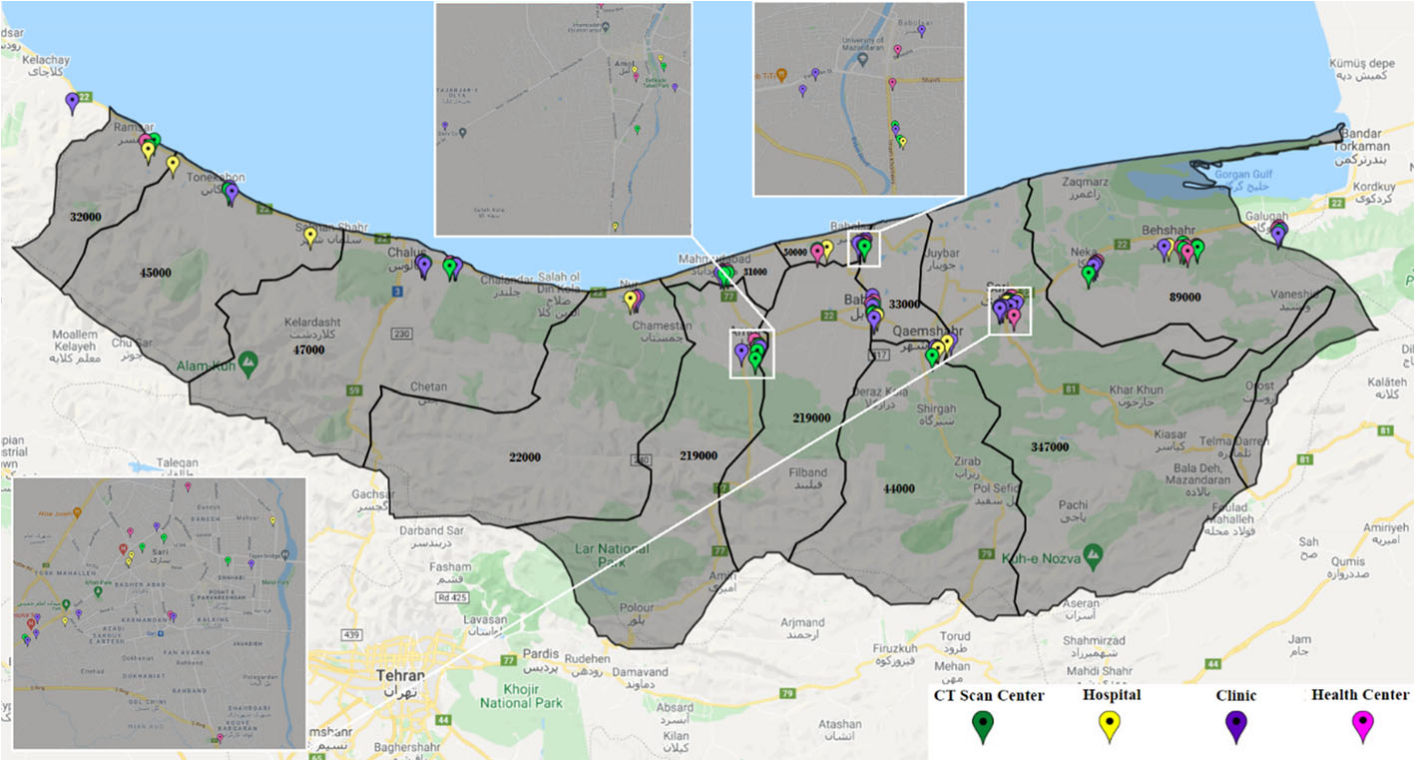
A schematic view of the facilities in the case study

As noted, only some health centers are activated to fight epidemic diseases. One of the main questions in our case is “which centers should be activated?” In this study, 30 health centers have been considered for initial tests. These centers use Coronavirus kits to test patients suspected of being infected. Additionally, there are 34 potential CT scan centers. In Mazandaran, 29 hospitals with different capacities and facilities can serve pandemic patients. The probabilities of improvement in each hospital vary due to their facilities and capacities. The 34 candidates for clinics in Mazandaran will also care for Coronavirus patients. These centers are privately run and care for Coronavirus patients at a relatively high cost. With the existence of the facilities, however, there is a possibility of higher recovery for patients. Figure 3 illustrates these centers on a Mazandaran map. Due to the small size of the cities of Neka and Galogah, they were merged with the city of Behshahr on the map, but their statistics are reported separately. Similarly, Fereydonkenar has been merged with Babolsar.

The government has provided funds to equip hospitals to provide better services for Coronavirus patients so that hospitals can procure necessary equipment for services. Therefore, the costs paid by these hospitals will be in the form of loan repayment, considering the present value of the loan, the interest rate, and the number of periods (Jahani et al., 2019).

The capacity of health centers, CT scans, hospitals, and clinics are reported in Tables 6, 7, 8, 9 in Appendix A. Tables 6 and 7show the probability of improvement in the hospital and clinic with respect to various risk statuses. The possibility of improvement is directly related to the facilities of medical centers, hospitals, and clinics. Tables A3 and A4 show the values of the probability of contracting the Coronavirus in various risk statuses. The capacity of health centers and CT scan centers are assumed to be constant during periods and as per any risk status. These parameters are collected in Tables 6 and 7. Hospitals and clinics have an initial capacity, and then in each period, according to the entry and exit of patients, their capacity is determined in subsequent periods. Therefore, the capacity of hospitals and clinics in the mathematical model of the problem is updated dynamically and only needs an initial capacity.

In this case, 21 different scenarios can occur, while the current situation is yellow. For instance, in Scenario 11, in the first period, the status is yellow, then in the subsequent periods, it is orange and red, respectively. In this study, the probability of occurrence for each scenario is considered equal.

### Computational results

**Fig. 4:**
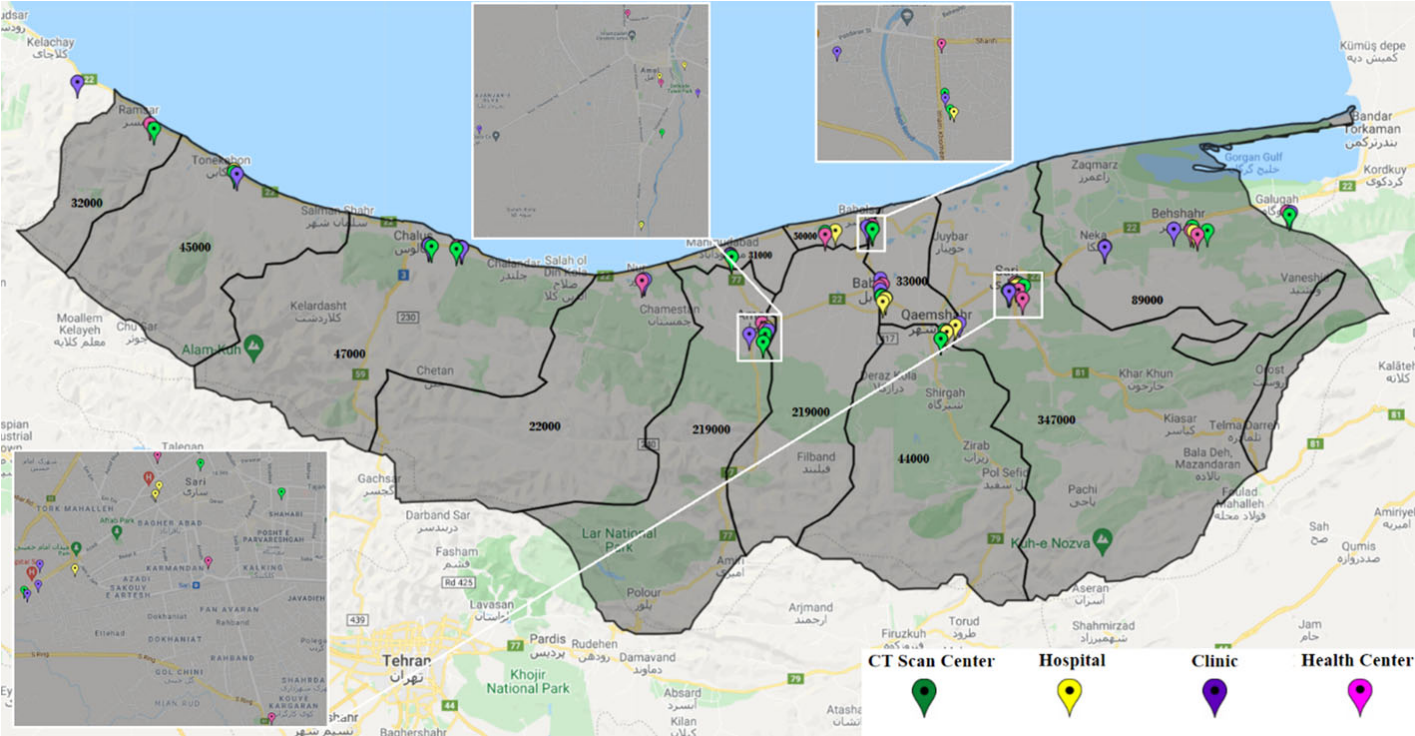
Case study: a general view of the result–activated centers

We run the model based on the above-mentioned input data, and accordingly, 22 health centers should be ready to operate. From the candidate CT scan centers, 24 should be activated. 19 hospitals and 20 clinics should also be activated. In different circumstances, various percentages of the hospitals’ capacities will be used. Figure 4 shows the healthcare network with activated centers according to the results of the model.

The values of the objective functions in a certain risk status and the multi-stage model (considering all scenarios with their probabilities) are shown in Table 1. The last column of the table demonstrates the aggregated objective function using LP metric.

**Table 1:** Case study: objective function values as per risk statuses

Objective function		First (improvement)	Second (cost of centers)	Third (mortality)
Certain	Blue status	173	8000	0.455
	Yellow status	164	8000	0.518
	Orange status	149	12,000	0.574
	Red status	125	14,000	0.665
Stochastic	Multi-stage	154	10,000	0.553

Once the status worsens (from blue to red status), the rate of improvement (the first objective function) decreases; i.e., the rate of infection increases. In the case of the second objective function, the cost of activating centers increases since we need more centers to be employed to serve Coronavirus patients. This trend is also seen in the probability of mortality, and the least amount of death occurs in the blue status. All these trends confirm the model’s applicability in the case of various risk levels.

**Fig. 5:**
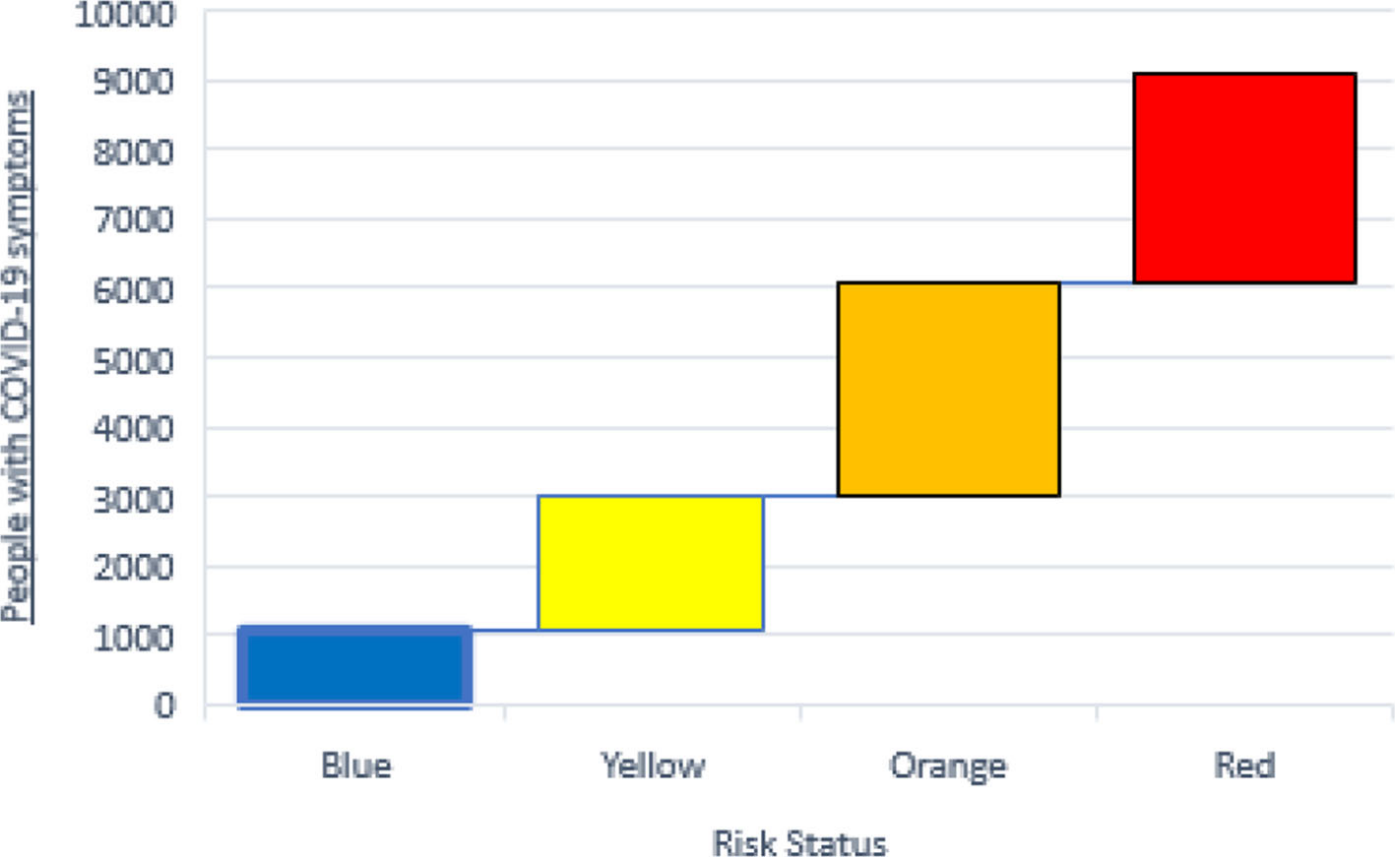
Case study: the definition of risk levels (statuses) from best to worst as per the total number of people with COVID‑19 symptoms

Each city of Mazandaran province can have one of the four colors, and according to the set of colors (risk levels), the color of the whole province will be determined. As shown in Fig. 5, the risk levels and their corresponding colors are determined by the total number of people suspected of having the disease in the province. Figure 5 shows that if the number of people suspected of having Coronavirus is less than 1,080 people in the whole province, the risk level turns blue. In this case, some cities in the province may be red or orange, but the whole province is still blue because the total number of people suspected of having the virus is the primary consideration. If the number of people is more than 1,080 but less than 3,020, Mazandaran province’s status will turn yellow, and so on for other risk levels.

**Fig. 6:**
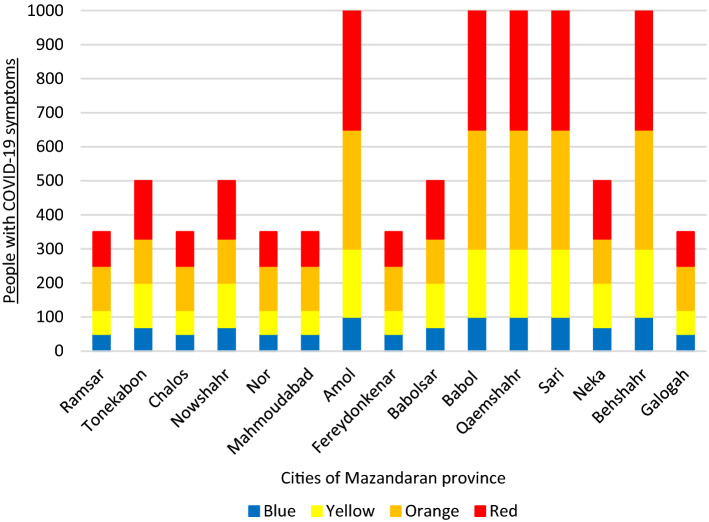
Case study: number of people with COVID‑19 symptoms in each city of Mazandaran province as per risk levels

Similar to what we have for the whole province, each city of the province will be at a particular risk level according to the range of people with Coronavirus symptoms. For instance, Ramsar will be blue if the number of people suspected of having the virus is less than 50, yellow if it is less than 120 and more than 50, orange if it is less than 250 and more than 120, and red if it is more than 250. If the amount is more than 350, it will be considered an emergency. These statistics will be 100, 300, 650, and 1,000 for Qaemshahr city, respectively, and 70, 200, 330, and 500 for Neka. Figure 6 illustrates the levels for each city.

**Fig. 7:**
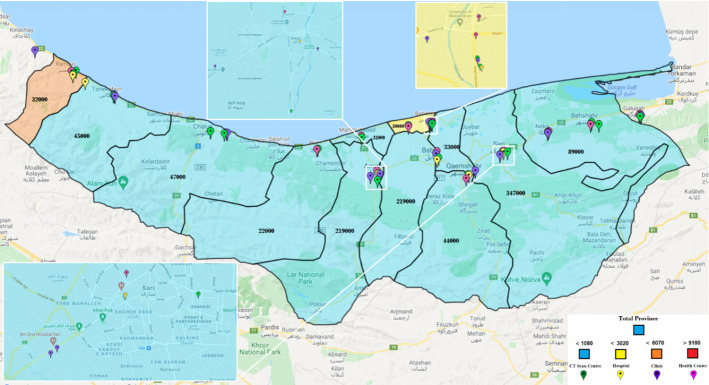
Case study: risk level statuses of cities in the case of the whole province being in blue status

In Fig. 7, we also investigate the situation that the color status of the whole province is normal (blue). In this case, the status of some cities has turned yellow and orange. The resultant number of people with COVID‑19 symptoms in each city is also reported in the figure. Table 10 in Appendix B shows the health centers activated in this case. The result demonstrates that out of 30 potential health centers, only 18 have been activated; out of 34 CT scan centers, 18 are activated; out of 29 hospitals, only 11 hospitals are activated; and out of 34 clinics, 14 clinics have been activated throughout the province.

Figure 9 illustrates the Mazandaran province once its status turns yellow. In this case, some cities take on other colors. Fereydunkenar has a red status, and Galogah, Nowshahr, Ghaemshahr, Babol, and Amol have an orange status. Neka, Babolsar, Mahmoudabad, Tonekabon and Ramsar are with blue status. This means that while only 4 of the 15 cities in the province are yellow, the color of the province as a whole is yellow.

**Fig. 8:**
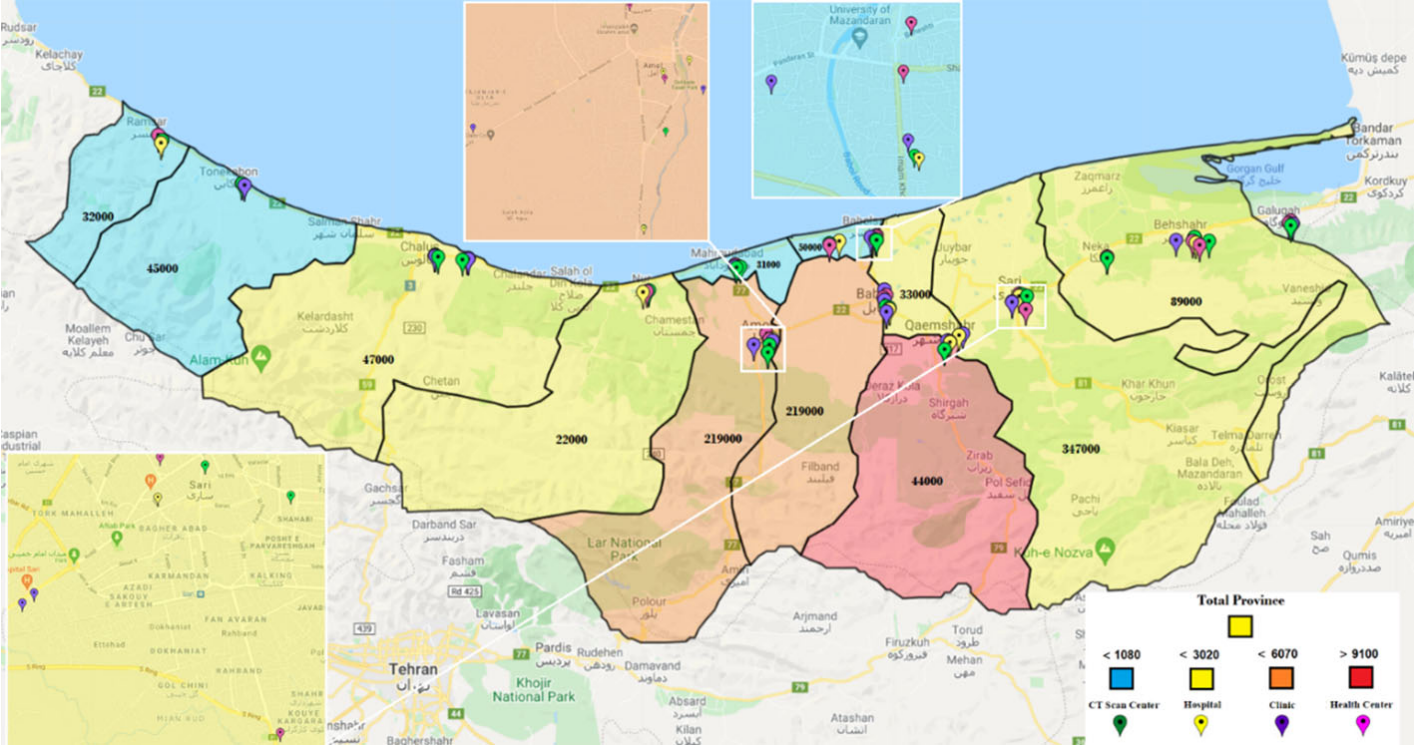
Case study: risk level statuses of cities in the case of the whole province being in yellow status

Table 11 reports the names of activated centers under Fig. 8. Accordingly, 22 health centers, 22 CT scan centers, 18 hospitals, and 19 clinics are activated.

**Fig. 9:**
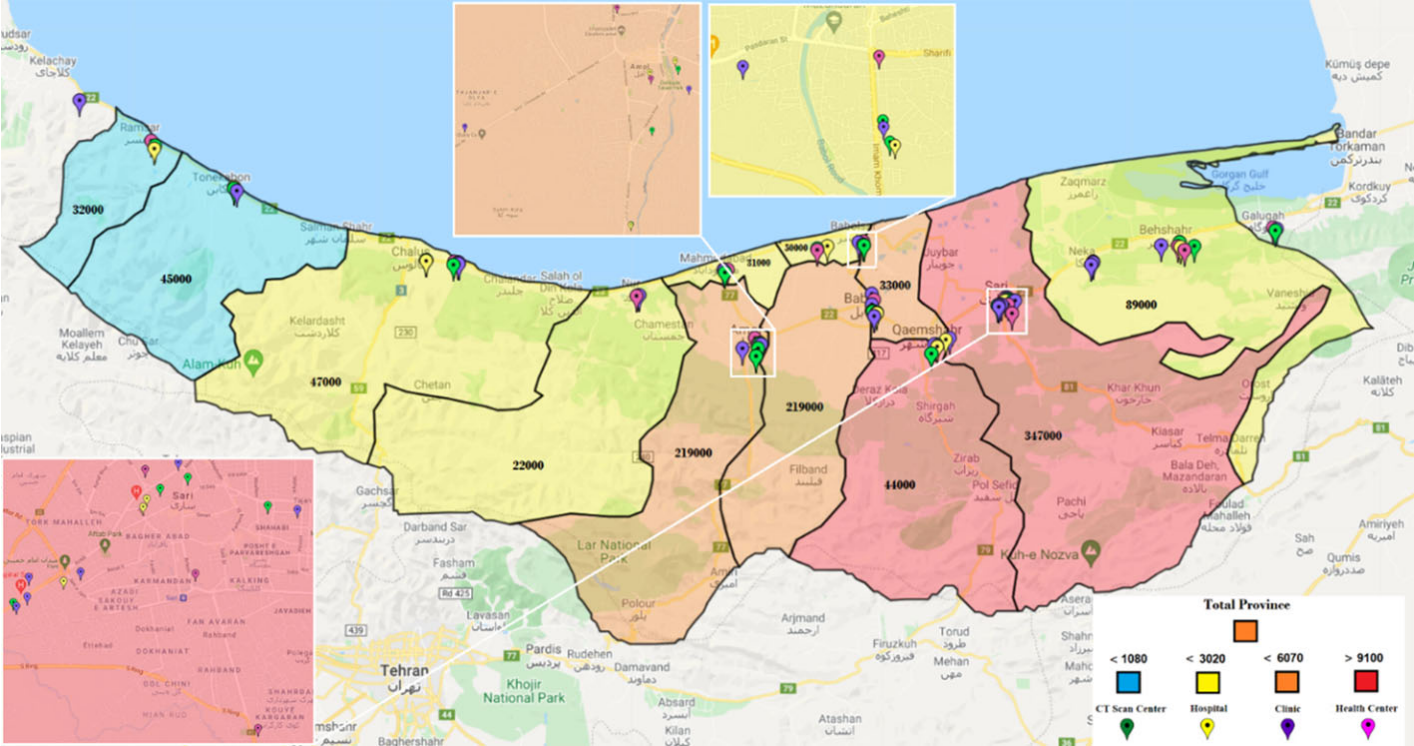
Case study: risk level statuses of cities in the case of the whole province being in orange status

Table 12 shows the activated centers under the orange status for the province, illustrated in Fig. 9. In this table, 22 health centers, 26 CT scan centers, 21 hospitals, and 25 clinics are active.

**Fig. 10:**
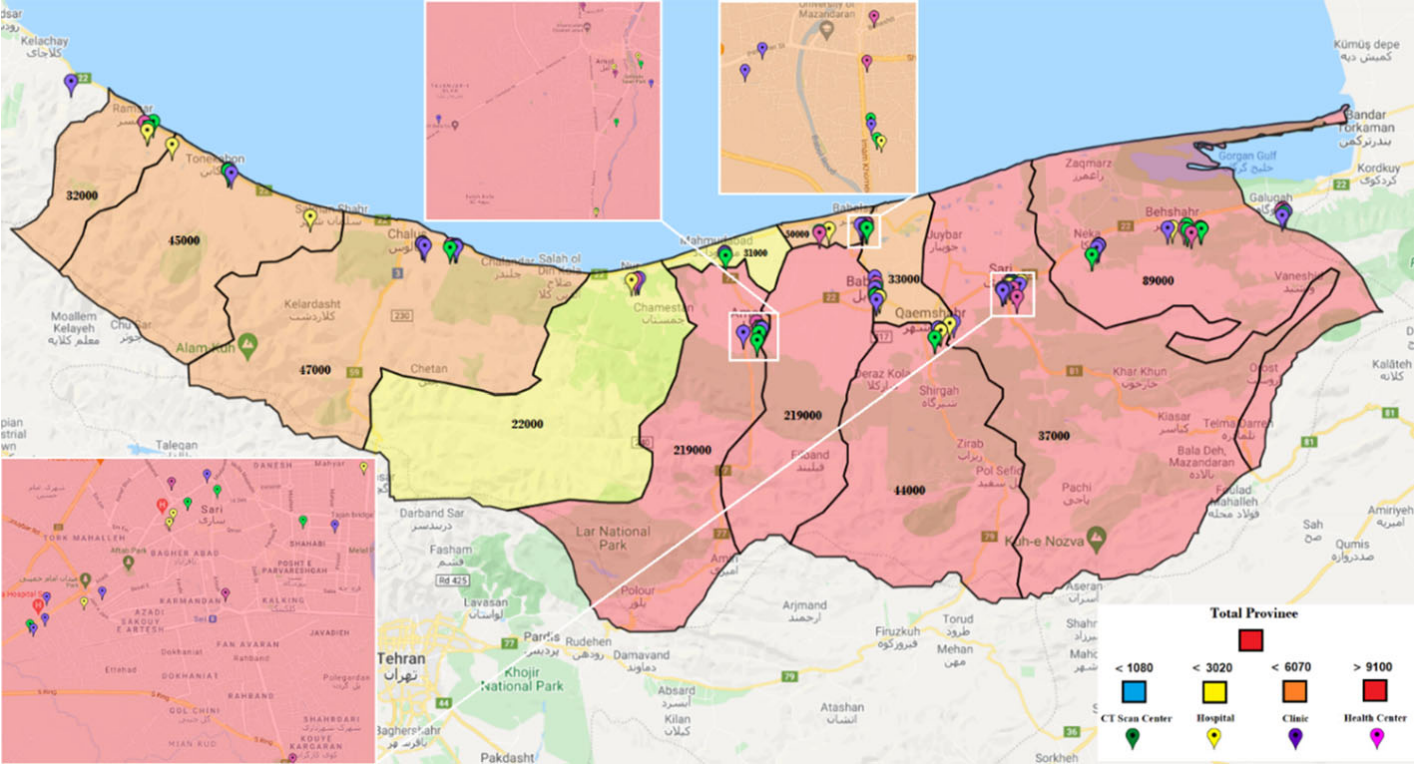
Case study: risk level statuses of cities in the case of the whole province being in red status

Table 13 and Fig. 10 reports the case of red status for the province, where 25 health centers, 32 CT scan centers, 29 hospitals, and 31 clinics are activated in the optimal solution.

**Fig. 11:**
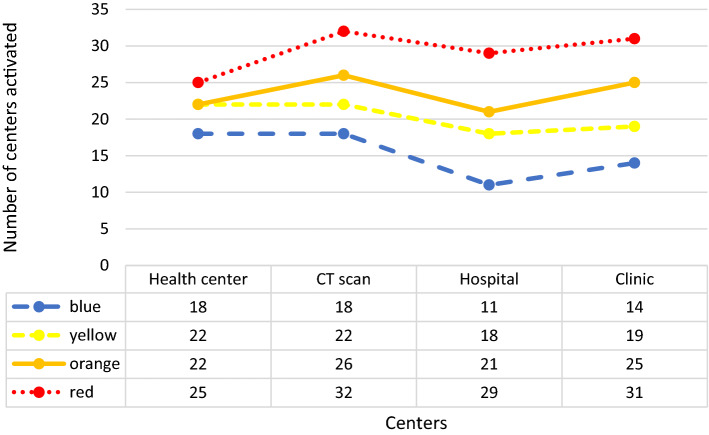
Number of activated centers considering various risk levels for the whole province

Figure 11 summarizes the above-mentioned results to highlight the required number of centers once the status changes from best to worst. The results show that although the total number of activated centers changes in higher proportions (from 61 to 117–91%), the total cost of activating the centers is controlled by the optimization model effectively in lower variations (from 8000 to 14,000 75%).

### Impacts of viable healthcare network design

**Fig. 12:**
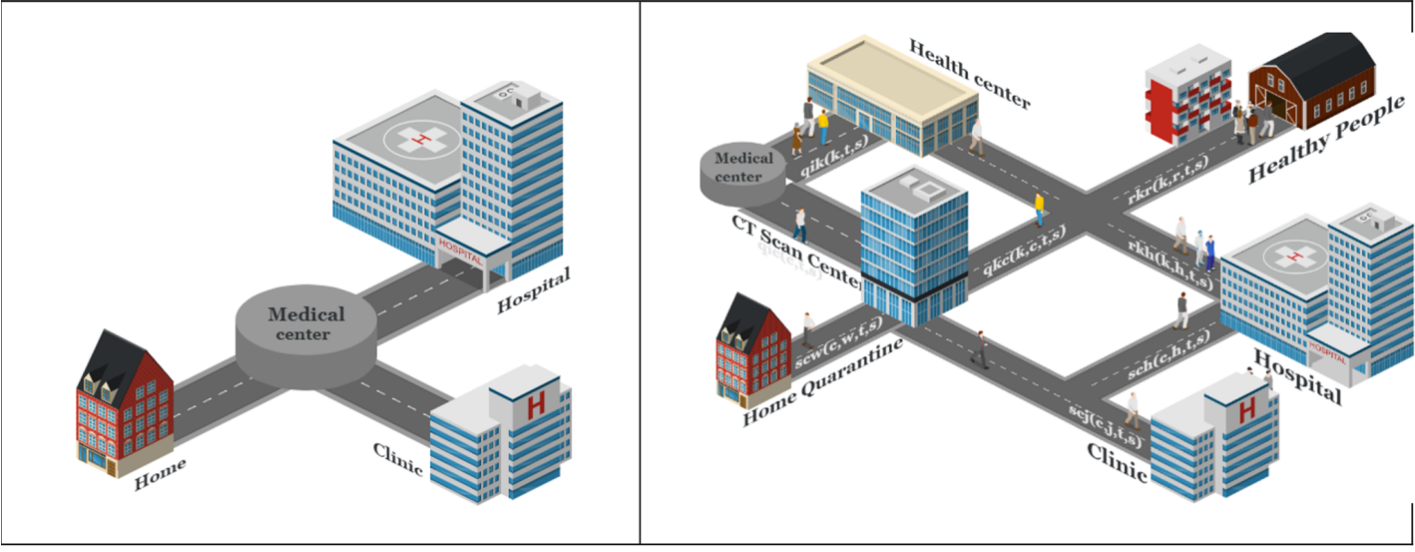
Schematic of a healthcare network in normal (left) and pandemic (right) situations

In a normal situation, when a pandemic does not exist, the healthcare network includes all health centers, hospitals, and clinics, and with a minimal number of people (or none at all) with Coronavirus symptoms. In this situation, patients are returned home according to the initial prescription of doctors in healthcare centers. Figure 12 illustrates the healthcare network in normal (left) and pandemic (right) situations. The differences in the grid are evident; the figure on the right is flexible for more tests on people suspected of being infected and provides initial control of the disease. We trust that the typical network will be able to cope with the next pandemic if the required changes for the pandemic network remain in place. For instance, the optimal capacity and facilities of the activated centers must be maintained even after the pandemic. This will be expensive for the system but will ensure that the system is viable for the next pandemic. The main question is this: How much will it cost to keep the healthcare system viable?

In this study, we only considered the costs of activating centers for serving Coronavirus patients. These costs include any extra facilities required for dealing with pandemic situations. To respond to the question mentioned above, we compare the extra cost imposed by the worst status (red) with the total annual cost of maintaining the healthcare system in normal situations. In our case study on Mazandaran province, we found this extra cost to be only 23% of the usual maintenance costs of the healthcare system. The COVID-19 crisis reminds every policymaker how underprepared the healthcare system was to detect and respond to emerging infectious diseases. Indeed, this small proportion of investment is worth the expense to keep the system viable. We also calculated this cost per person (by dividing it by the population of the province) and found that only $4 per person per year can assist to guarantee far better preparation for a future epidemic.

### Numerical examples

In this step, we solve the problem for several network sizes by generating numerical data from our real case study. We name these sets as small, medium, and large to examine the efficiency of the solution methodology concerning various problem sizes. Tables 2, 3, and 4 report the results of the problem sets in small, medium, and large dimensions, respectively. The numbers of health centers, CT scan centers, hospitals, and clinics appear at the top of each table. These numbers are logically increased according to the real problems that occur in larger provinces/countries. Our case study of Mazandaran province is classified as medium size. However, in the large size problem (Table 4), we assume a network with a population ten times that of Mazandaran. For an example of such a large network, we assume the entire country of Iran, with Mazandaran as one of its cities.

**Table 2:** Case study: results of the model considering a small size problem

Dimension	Index		K = 2, C = 2, H = 3, J = 2, T = 4, W = 1, R = 1		
	Objective function		First (improvement)	Second (cost of centers)	Third (mortality)
Small size	Certain	Blue status	14.3	20,040	0.525
		Yellow status	11.8	20,040	0.570
		Orange status	10.9	24,010	0.641
		Red status	10.1	28,080	0.692
	Stochastic	Multi-stage	11.6	23,040	0.602

**Table 3:** Case study: Results of the model considering a small-medium problem

Dimension	Index		K = 9, C = 6, H = 16, J = 9, T = 4, W = 1,R = 1		
	Objective function		First (improvement)	Second (cost of centers)	Third (mortality)
Medium size	Certain	Blue status	69.1	55,600	0.561
		Yellow status	63.9	56,000	0.598
		Orange status	54.8	58,080	0.663
		Red status	49.2	52,000	0.689
	Stochastic	Multi-stage	59.6	57,200	0.619

**Table 4:** Case study: results of the model considering a large size problem

Dimension	Index		K = 200, C = 99, H = 330, J = 250, T = 4, W = 1, R = 1		
	Objective function		First (improvement)	Second (cost of centers)	Third (mortality)
Large size	Certain	Blue status	328.9	140,300	0.402
		Yellow status	277.3	160,020	0.441
		Orange status	261.6	190,000	0.494
		Red status	232.3	250,000	0.567
	Stochastic	Multi-stage	270.4	180,200	0.473

As the results of all tables show, the model can solve all proposed problems—from small to large sizes—in reasonable computational times. In all cases, the results of the multi-stage stochastic model are a value between the results of the yellow and orange statuses. This also confirms the efficiency of the stochastic solution with the consideration of probable scenarios.

Comparing the objective function values reveals that although the large networks impose greater costs (the second objective function) as more centers are required for activation, in these network problem sizes, the model efficiently increases the improvement rates (the first objective function) and decreases the probability of death (the third objective function).

## Sensitivity analyses and managerial implications

**Fig. 13:**
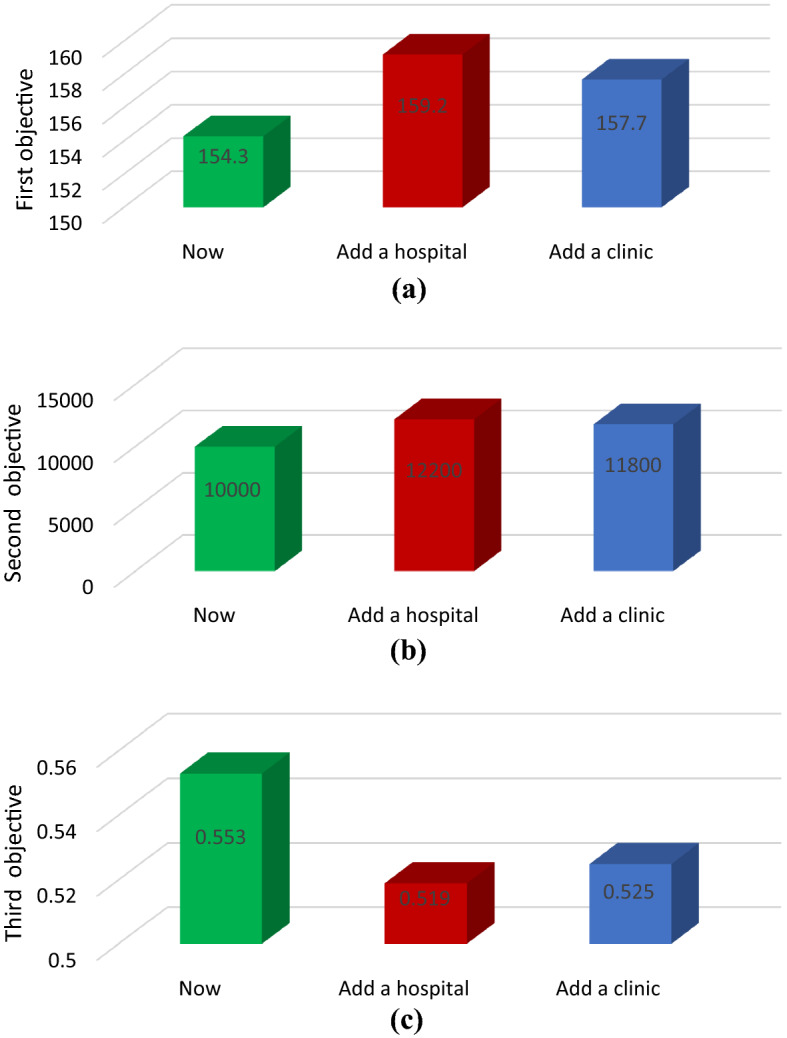
Effect of adding a hospital or clinic on the objective functions of the model

In any mathematical model, investigating the crucial parameters of the model is of utmost importance to discuss several managerial insights. Even small changes in the initial parameters can have a significant impact on the results (objective functions). Managers/organizers need to know the impact of each parameter that has special considerations for them. In our proposed problem, if the number of patients with Coronavirus symptoms entering the centers increases by 10%, then the capacity of some candidate hospitals might not be large enough. So, the network may need more candidate centers added to the model to cope with the issue. An important question here is if a hospital is added to the network for Coronavirus patients, then how will the objectives change? Fig. 13 shows the effect of adding a hospital or a clinic to the network for the first (part a), second (part b), and third (part c) objective functions. In this case, the first objective function (the probability of improvement) increases by 5%, the second objective function (the cost of activated centers) increases by 9%, and the third objective function (mortality rate) is reduced by 6%. On the other hand, adding a clinic will lead to a 4% increase, an 11% increase, and a 5% decrease in target values, respectively. Comparing these effects will give us the insight that adding a hospital to the list of those accepting Coronavirus patients will result in better improvement and decreasing the probability of mortality than a clinic. However, the cost of preparing the hospital is higher.

**Fig. 14:**
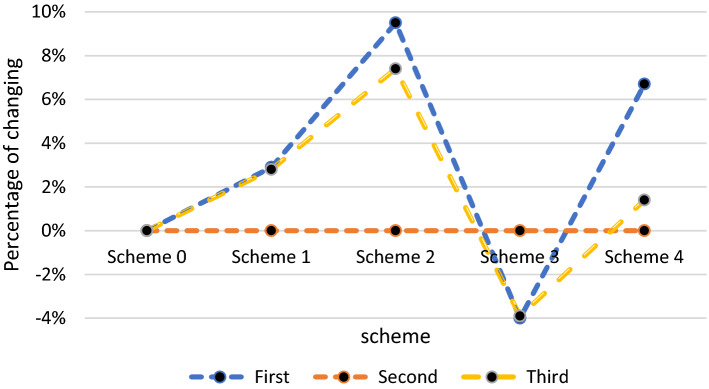
The effect of hospital and clinic input parameters on objectives

Another analysis considers various schemes in which the rate of change in the probability of mortality is investigated in the third objective function based on increasing admission rates to hospitals and clinics. Scheme 0 is defined as the current state of the problem. Table 5 introduces all schemes concerning the changes in the admission rates. For instance, Scheme 1 is defined by a 5% increase in the rate of hospital admission through health centers, and at the same time, a 5% decrease in the number of people whose tests are negative. As shown in Fig. 14, all the assumed changes in admission rates do not alter the total costs of activating centers. However, the rate of improvement and the probability of mortality would change significantly. In Scheme 2, the changes are more considerable due to the importance of the rate of admission to the hospital through the CT scan center and the rate of transfer to quarantine compared to the previous scheme. In other words, the rate of admission to the hospital through the CT scan center will increase by 5%, and the rate of transfer to quarantine will decrease by 5%. The adverse effects gained in Scheme 3 are justified by reducing 10 and 5 percentages in the rate of admission to hospitals through health centers and CT scan centers. Additionally, the rate of admission to clinics was reduced by 5%, transfer to home quarantines was reduced by 10%, and return to the community was increased by 10%.

**Table 5:** Case study: all schemes

Objective function	First (improvement)	Second (cost of centers)	Third (mortality)
Scheme 1	0.029	0	0.028
Scheme 2	0.095	0	0.074
Scheme 3	− 0.04	0	− 0.039
Scheme 4	0.067	0	0.014

**Fig. 15:**
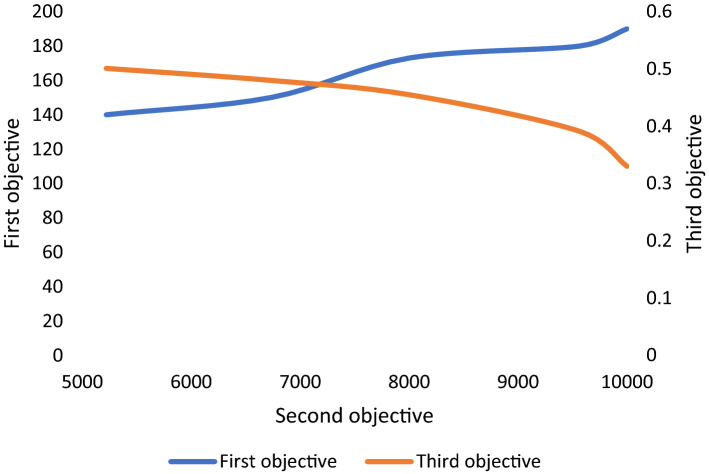
Trade-off among the first and third objectives of the proposed model

We also investigate the relations between the defined objective functions. We realized that once the second objective function (cost) increases, so does the first objective function (recovery rate of patients). On the other hand, as shown in Fig. 15, with the same proportion of increase in the second objective function, the third objective function (mortality rate of patients) decreases. This conflict between these two objectives were expected and confirmed in this test.

Besides the preceding insights, this study provides valuable suggestions for managers from different perspectives. Considering the findings, some of the recommendations are:

- Under current circumstances, when there is no pandemic, the healthcare network must always be watchful for an alarm. Under normal conditions, hospitals are ready to provide services to those who need them, but in emergencies, this simply cannot happen. If a hospital is added to the problem in this situation, although it will cost an additional 22%, it will contribute approximately 3% to the improvement of patients. This amount is thought-provoking and valuable. Furthermore, it reduces the probability of mortality by 6%, based on the third objective.
- Input parameters are highly sensitive. The likelihood of patients recovering in hospitals and clinics is based on past observations and depends on factors such as medical equipment and specialist staffing. The death toll of a pandemic has always been high, and saving the lives of every human is essential, so managers must strive to increase the likelihood of improving patients’ health.
- Another parameter that can directly impact lowering the number of patients is the probability of infection. This parameter depends on various factors such as the decontamination of centers, the number of patients referred, the number of workers, and others. Special consideration is required for these related factors to keep the probability as low as possible.
- The capacity of centers is always a crucial parameter in the healthcare network, especially during a pandemic. Managers of centers should be concerned about dynamically controlling the capacities, which can only happen via a detailed plan like our model.
- The time horizon of the plan is also an important parameter for the model because it reflects the effects of decisions during time periods. In healthcare, periods are not too long, and when a pandemic is involved, they are even shorter. Therefore, managers should be prepared to make strategic decisions in short periods of time.

## Conclusion

The healthcare supply chain network in every country or area should be defined so that when a pandemic occurs, it will function well to save human lives. In this study, considering the COVID-19 pandemic, an attempt has been made to design a viable healthcare supply chain network. With a review of previous research, it has been observed that the design of the healthcare supply chain network has received less attention. On the other hand, the COVID-19 pandemic has provided extensive and up-to-date information that can be used for the design. In different countries, according to the severity and weakness of the virus’s prevalence, several risk levels have been defined and illustrated by specific colors. In this study, four colors are considered: blue, yellow, orange, and red, from natural to emergency statuses, respectively. We consider a healthcare supply chain network with the objectives of maximizing the probability of patients recovering and reducing the risk of infection, minimizing the costs of activating centers for serving the patients of the Coronavirus, and minimizing the possibility of death for patients admitted to hospitals. The proposed problem considered different stages. Once people are suspected of having the disease, they go to health centers or CT scan centers. If their test results are negative in the health center, they return to the community, but if their results are positive, they go to a hospital or a CT scan center. After being referred to a CT scan center, they are transferred to home quarantine, a hospital, or a clinic per the advice of a specialist, depending on the extent of their involvement with the disease. In the proposed problem, the capacity of each center can be updated dynamically in each period. A multi-stage stochastic programming approach is used in which the risk level in each stage can remain unchanged, become better, or get worse compared to the previous stage. An LP methodology is employed for solving the proposed multi-objective model.

By implementing the model in a real case study from the Mazandaran province in Iran, it was demonstrated how many of the candidate centers should be activated to have an optimal network. By presenting the results of the problem and developing several sensitivity analyses, different angles of the problem and the effectiveness of the healthcare network were investigated. By developing several numerical examples from the real case study data, various dimensions of the problem set (small, medium, and large) were examined to demonstrate the model’s applicability. Several managerial insights were also presented.

As future directions for the study, we suggest that the demand (number of patients) be considered as an uncertain parameter. This research can also be examined from a scheduling perspective. This approach is highly recommended because time is of the essence in improving patients’ health networks. Since it seems that the Coronavirus wave will not be interrupted and will continue even when weak, it is imperative to carefully examine the disruption that infectious diseases can bring to the health supply chain. A general concept about the ripple effect of infectious disruption can be examined.

## Appendix A: case study input data

See Tables 6, 7, 8, 9.

**Table 6:** Case study: probability of improvement and capacity of hospitals

#	Name	City	Capacity	Probability of improvement in risk level			
				Blue	Yellow	Orange	Red
1	Imam sajjad	Ramsar	600	0.7	0.66	0.6	0.6
2	Ahmadnejad	Ramsar	650	0.65	0.6	0.6	0.6
3	Rajaei	Tonekabon	650	0.72	0.7	0.68	0.64
4	Abbasabad	Tonekabon	550	0.68	0.65	0.63	0.61
5	Taleqani	Chalos	650	0.75	0.71	0.65	0.62
6	Nowshahr	Nowshahr	600	0.75	0.75	0.73	0.7
7	Imam Khomeini	Nor	580	0.65	0.64	0.61	0.6
8	Shohada	Mahmoudabad	650	0.68	0.65	0.6	0.6
9	17 Shahrivar	Amol	650	0.85	0.8	0.77	0.75
10	Imam Reza	Amol	650	0.7	0.68	0.65	0.63
11	Shomal	Amol	700	0.78	0.63	0.61	0.61
12	Imam Khomeini	Fereydonkenar	650	0.68	0.63	0.62	0.6
13	Shafa	Babolsar	700	0.75	0.65	0.63	0.61
14	Rouhani	Babol	650	0.78	0.7	0.68	0.65
15	Shahid Beheshti	Babol	650	0.75	0.7	0.7	0.68
16	Yahyanejad	Babol	500	0.75	0.66	0.65	0.6
17	Razi	Qaemshahr	800	0.75	0.7	0.69	0.64
18	Valieasr	Qaemshahr	700	0.75	0.65	0.64	0.6
19	Shafa	Sari	620	0.8	0.73	0.7	0.65
20	Imam Khomeini	Sari	800	0.8	0.68	0.65	0.61
21	Baghiyat Allah	Sari	600	0.75	0.6	0.61	0.6
22	Amir mazandarani	Sari	750	0.7	0.65	0.62	0.6
23	Imam Hossein	Neka	700	0.75	0.66	0.63	0.62
24	Bou Ali Sina	Neka	600	0.78	0.63	0.6	0.6
25	Mehr	Behshahr	700	0.75	0.7	0.68	0.66
26	Imam Khomeini	Behshahr	800	0.78	0.7	0.7	0.68
27	Khatam Al-Anbia	Behshahr	550	0.7	0.6	0.6	0.6
28	Shohada	Behshahr	550	0.75	0.65	0.63	0.61
29	Samenol Ammeh	Galogah	600	0.78	0.6	0.6	0.6

**Table 7:** Case study: probability of improvement and capacity of clinics

#	Name	City	Capacity	Probability of improvement in risk level			
				Blue	Yellow	Orange	Red
1	Clinic 1	Nor	150	0.95	0.93	0.91	0.87
2	Imam Reza	Ramsar	250	0.94	0.92	0.9	0.84
3	Clinic 1	Tonekabon	250	0.96	0.95	0.92	0.86
4	Clinic 2	Tonekabon	135	0.92	0.92	0.91	0.88
5	Iran clinic	Nowshahr	150	0.96	0.95	0.92	0.89
6	Imam Reza	Nowshahr	250	0.95	0.93	0.91	0.86
7	Mehr	Nowshahr	130	0.92	0.91	0.9	0.88
8	Sina	Nowshahr	240	0.97	0.96	0.92	0.89
9	Helal Ahmar	Mahmoudabad	235	0.92	0.91	0.88	0.82
10	Shomal	Amol	245	0.92	0.92	0.88	0.79
11	Nick darman	Amol	160	0.97	0.95	0.92	0.84
12	Imam Ali	Amol	150	0.93	0.92	0.89	0.8
13	Tamin Ejtemai	Mahmoudabad	150	0.91	0.9	0.86	0.81
14	Shahid salehi	Babolsar	160	0.92	0.91	0.89	0.82
15	Babol clinic	Babol	260	0.97	0.96	0.92	0.86
16	Mehregan	Babol	245	0.95	0.92	0.9	0.8
17	Omid	Babol	115	0.92	0.91	0.86	0.76
18	Boarding Clinic Alborz	Qaemshahr	135	0.92	0.9	0.86	0.79
19	Ghaem	Qaemshahr	245	0.94	0.91	0.89	0.79
20	Ibn Sina	Sari	255	0.96	0.93	0.9	0.84
21	Hekmat	Sari	135	0.92	0.91	0.88	0.81
22	Fatemeh Zahra	Sari	115	0.91	0.9	0.86	0.8
23	Nime shaban	Sari	250	0.92	0.9	0.85	0.8
24	Vali Asr	Sari	245	0.91	0.91	0.88	0.81
25	Velaiat	Sari	115	0.91	0.9	0.85	0.81
26	Noor	Sari	110	0.91	0.91	0.84	0.78
27	Nime shaban	Neka	250	0.94	0.91	0.88	0.79
28	Farhangian	Neka	140	0.92	0.91	0.87	0.78
29	Darmangah	Behshahr	160	0.95	0.93	0.89	0.81
30	Sepid jamegan	Babolsar	145	0.95	0.93	0.9	0.85
31	Arian	Babolsar	150	0.96	0.94	0.9	0.85
32	Kian	Babolsar	120	0.92	0.91	0.86	0.81
33	Galogah clinic	Galogah	250	0.95	0.93	0.89	0.8
34	Samen	Galogah	145	0.95	0.94	0.88	0.8

**Table 8:** Case study: Probability of infection and capacity of health centers

#	Name	City	Capacity	Probability of infection in risk level			
				Blue	Yellow	Orange	Red
1	Health center 1	Nor	800	0.2	0.2	0.22	0.3
2	Health center 1	Ramsar	950	0.2	0.25	0.27	0.36
3	Health center 1	Chalos	800	0.22	0.25	0.29	0.37
4	Health center 2	Chalos	950	0.35	0.4	0.4	0.44
5	Health center 1	Nowshahr	900	0.23	0.3	0.35	0.4
6	Health center 2	Nowshahr	850	0.3	0.35	0.38	0.43
7	Health center 1	Tonekabon	900	0.2	0.2	0.24	0.44
8	Health center 1	Fereydonkenar	750	0.3	0.35	0.36	0.42
9	Health center 1	Mahmoudabad	800	0.21	0.25	0.28	0.33
10	Health center 1	Amol	900	0.15	0.2	0.28	0.36
11	Health center 2	Amol	1000	0.2	0.25	0.29	0.37
12	Health center 1	Galogah	950	0.19	0.25	0.3	0.35
13	Health center 2	Mahmoudabad	800	0.3	0.4	0.43	0.48
14	Health center 1	Babolsar	950	0.15	0.2	0.26	0.32
15	Health center 1	Babol	900	0.17	0.2	0.27	0.37
16	Health center 2	Babol	900	0.18	0.25	0.24	0.3
17	Health center 2	Galogah	500	0.33	0.4	0.45	0.49
18	Health center 1	Qaemshahr	800	0.15	0.2	0.24	0.31
19	Health center 2	Qaemshahr	900	0.2	0.25	0.31	0.38
20	Health center 1	Sari	1000	0.15	0.2	0.28	0.32
21	Health center 2	Sari	900	0.18	0.2	0.25	0.33
22	Health center 3	Sari	800	0.17	0.25	0.33	0.38
23	Health center 4	Sari	500	0.25	0.3	0.33	0.39
24	Health center 1	Behshahr	900	0.2	0.25	0.29	0.37
25	Health center 2	Behshahr	850	0.18	0.25	0.3	0.4
26	Health center 3	Behshahr	600	0.22	0.35	0.37	0.41
27	Health center 1	Neka	700	0.26	0.3	0.35	0.4
28	Health center 2	Neka	600	0.27	0.35	0.39	0.45
29	Health center 4	Behshahr	800	0.21	0.25	0.32	0.38
30	Health center 2	Babolsar	750	0.2	0.25	0.3	0.34

**Table 9:** Case study: probability of infection and capacity of CT scan centers

*#*	Name	City	Capacity	Probability of infection in risk level			
				Blue	Yellow	Orange	Red
1	CT scan 1	Nor	900	0.3	0.3	0.33	0.39
2	CT scan 1	Ramsar	950	0.35	0.35	0.36	0.39
3	CT scan 1	Chalos	900	0.28	0.3	0.33	0.39
4	CT scan 2	Chalos	950	0.33	0.4	0.42	0.46
5	CT scan 1	Nowshahr	800	0.3	0.35	0.38	0.42
6	CT scan 2	Nowshahr	950	0.3	0.33	0.33	0.37
7	CT scan 1	Tonekabon	1000	0.29	0.3	0.31	0.35
8	CT scan 1	Fereydonkenar	950	0.3	0.35	0.37	0.39
9	CT scan 1	Mahmoudabad	900	0.3	0.36	0.39	0.41
10	CT scan 1	Amol	1000	0.27	0.33	0.39	0.44
11	CT scan 2	Amol	1100	0.27	0.31	0.35	0.4
12	CT scan 1	Galogah	950	0.25	0.3	0.36	0.42
13	CT scan 2	Mahmoudabad	700	0.3	0.34	0.38	0.44
14	CT scan 1	Babolsar	950	0.28	0.31	0.32	0.38
15	CT scan 1	Babol	950	0.26	0.3	0.36	0.39
16	CT scan 2	Babol	950	0.3	0.35	0.38	0.45
17	CT scan 2	Ramsar	600	0.3	0.33	0.39	0.46
18	CT scan 1	Qaemshahr	900	0.27	0.32	0.36	0.4
19	CT scan 2	Qaemshahr	900	0.29	0.31	0.38	0.44
20	CT scan 1	Sari	950	0.25	0.3	0.39	0.45
21	CT scan 2	Sari	850	0.25	0.3	0.35	0.44
22	CT scan 3	Sari	850	0.3	0.35	0.4	0.43
23	CT scan 4	Sari	800	0.29	0.31	0.37	0.4
24	CT scan 1	Behshahr	700	0.32	0.35	0.39	0.4
25	CT scan 2	Behshahr	750	0.29	0.31	0.34	0.43
26	CT scan 3	Behshahr	800	0.3	0.33	0.38	0.44
27	CT scan 1	Neka	600	0.29	0.31	0.38	0.43
28	CT scan 2	Neka	500	0.29	0.35	0.39	0.44
29	CT scan 3	Amol	900	0.27	0.3	0.33	0.37
30	CT scan 2	Babolsar	850	0.3	0.35	0.38	0.39
31	CT scan 3	Babol	850	0.3	0.33	0.36	0.37
32	CT scan 4	Babol	800	0.26	0.31	0.35	0.37
33	CT scan 3	Qaemshahr	650	0.26	0.3	0.33	0.36
34	CT scan 4	Qaemshahr	600	0.3	0.35	0.36	0.38

## Appendix B: case study results

See Tables 10, 11,12, 13.

**Table 10:** Case study: activated centers in the case of blue risk status for the whole province

*#*	Health center	CT scan	Hospital	Clinic
1	Ramsar health center 1	CT scan Babol 1	Imam Sajjad	Babol clinic
2	Tonekabon health center 1	CT scan Ramsar 1	Ahmadnejad	Ghaem clinic
3	Chalos health center 1	CT scan Ramsar 2	Taleqani	Ibn sina
4	Nowshahr health center 1	CT scan Babolsar 1	17 Shahrivar	Nime Shaban Sari
5	Nor health center 1	CT scan Babolsar 2	Shafa	Nime Shaban Neka
6	Mahmoudabad health center 1	CT scan Tonekabon 1	Rouhani	Samen
7	Amol health center 1	CT scan Chalos 1	Razi	Shahid Salehi
8	Babolsar health center 1	CT scan Behshahr 1	Imam Khomeini	Arian
9	Babolsar health center 2	CT scan Behshahr 3	Imam Hossein	Sina
10	Babol health center 1	CT scan Nowshahr 1	Imam Khomeini	Nick Darman
11	Qaemshahr health center 1	CT scan Nor 1	Samenol Ammeh	Imam Ali
12	Sari health center 1	CT scan Fereydonkenar 1		Ramsar clinic
13	Neka health center 1	CT scan Mahmoudabad 1		Tonekabon 1 clinic
14	Neka health center 2	CT scan Amol 1		Tonekabon 2 clinic
15	Behshahr health center 1	CT scan Sari 1		
16	Galogah health center 1	CT scan Qaemshahr 1		
17	Fereydonkenar health center 1	CT scan Neka 1		
18	Galogah health center 2	CT scan Galogah 1

**Table 11:** Case study: activated centers in the case of yellow risk status for the whole province

#	Health center	CT scan	Hospital	Clinic
1	Ramsar health center 1	CT scan Babol 1	Razi	Babol clinic
2	Tonekabon health center 1	CT scan Ramsar 1	Valiasser	Ghaem clinic
3	Chalos health center 1	CT scan Behshahr 3	Shahid Beheshti	Ibn Sina
4	Nowshahr health center 1	CT scan Babolsar 1	Rouhani	Nime Shaban Sari
5	Nor health center 1	CT scan Sari 2	Shafa Babolsar	Mehregan
6	Mahmoudabad health center 1	CT scan Tonekabon 1	Emam Khomeini sari	Samen
7	Amol health center 1	CT scan Chalos 1	Taleqani	Shahid Salehi
8	Babolsar health center 1	CT scan Behshahr 1	17 Shahrivar	Arian
9	Babolsar health center 2	CT scan Chalos 2	Imam Reza	Sina
10	Babol health center 1	CT scan Nowshahr 1	Shomal	Nick Darman
11	Qaemshahr health center 1	CT scan Noor 1	Imam Hossein	Omid
12	Sari health center 1	CT scan Fereydonkenar 1	Mehr	Boarding Clinic Alborz
13	Neka health center 1	CT scan Mahmoudabad 1	Emam Khomeini Behshahr	Tonekabon 1 clinic
14	Sari health center 2	CT scan Amol 1	Samenol Ammeh	Darmongah Behshahr
15	Behshahr health center 1	CT scan Sari 1	Nowshahr	Tamin Ejtemai
16	Galogah health center 1	CT scan Qaemshahr 1	Imam Sajjad	Iran Clinic
17	Fereydonkenar health center 1	CT scan Neka 1	Emam Khomeini Nor	Mehr
18	Galogah health center 2	CT scan Galogah 1	Emam Khomeini Fereydonkenar	Shomal
19	Babol health center 2	CT scan Qaemshahr 3		Imam Ali
20	Amol health center 2	CT scan Amol 2		
21	Behshahr health center 2	CT scan Babol 3		
22	Qaemshahr health center 2	CT scan Mahmoudabad 2

**Table 12:** Case study: Activated centers in the case of orange risk status for the whole province

	Health center	CT scan	Hospital	Clinic
1	Ramsar health center 1	CT scan Babol 1	Razi	Babol clinic
2	Tonekabon health center 1	CT scan Ramsar 1	Valiasser	Ghaem clinic
3	Chalos health center 1	CT scan Babol 2	Shahid Beheshti	Ibn Sina
4	Nowshahr health center 1	CT scan Babolsar 1	Rouhani	Nime Shaban Sari
5	Nor health center 1	CT scan Babol 3	Yahya Nejad	Farhangian
6	Mahmoudabad health center 1	CT scan Tonekabon 1	Shafa Babolsar	Samen
7	Amol health center 1	CT scan Chalos 1	Emam Khomeini Sari	Shahid Salehi
8	Babolsar health center 1	CT scan Behshahr 1	Baghiyat Allah	Arian
9	Qaemshahr health center 2	CT scan Qaemshahr 3	Amir Mazandarani	Tamin Ejtemai
10	Babol health center 1	CT scan Nowshahr 1	Shohada	Darmangah
11	Qaemshahr health center 1	CT scan Nor 1	Taleqani	Iran clinic
12	Sari health center 1	CT scan Fereydonkenar 1	17 Shahrivar	Ramsar clinic
13	Neka health center 1	CT scan Mahmoudabad 1	Imam Reza	Tonekabon 1 clinic
14	Sari health center 2	CT scan Amol 1	Shomal	Nor 1 clinic
15	Behshahr health center 1	CT scan Sari 1	Imam Hossein	Sina clinic
16	Galogah health center 1	CT scan Qaemshahr 1	Mehr	Mehregan
17	Fereydonkenar health center 1	CT scan Neka 1	Emam Khomeini Behshahr	Omid
18	Sari health center 3	CT scan Galogah 1	Nowshahr	Boarding Clinic Alborz
19	Behshahr health center 4	CT scan Qaemshahr 4	Imam sajjad	Hekmat
20	Amol health center 2	CT scan Sari 2	Rajaei	Vali Asr
21	Mahmoudabad health center 2	CT scan Sari 3	Emam Khomeini fereydonkenar	Velaiat
22	Nowshahr health center 2	CT scan Sari 4		Noor
23		CT scan Behshahr 3		Nime shaban Neka
24		CT scan Babolsar 2		Imam Ali
25		CT scan Amol 2		Nick Darman
26		CT scan Amol 3

**Table 13:** Case study: Activated centers in the case of red risk status for the whole province

#	Health center	CT scan	Hospital	Clinic
1	Ramsar health center 1	CT scan babol 1	Razi	Babol clinic
2	Tonekabon health center 1	CT scan Ramsar 1	Valiasser	Ghaem clinic
3	Chalos health center 1	CT scan Babol 2	Shahid Beheshti	Ibn Sina
4	Nowshahr health center 1	CT scan Babolsar 1	Rouhani	Nime Shaban Sari
5	Nor health center 1	CT scan Babol 3	Yahya Nejad	Nime Shaban Neka
6	Mahmoudabad health center 1	CT scan Tonekabon 1	Shafa Babolsar	Samen
7	Amol health center 1	CT scan Chalos 1	Emam Khomeini Sari	Shahid Salehi
8	Babolsar health center 1	CT scan Behshahr 1	Baghiyat Allah	Arian
9	Qaemshahr health center 2	CT scan Babol 4	Amir Mazandarani	Sina
10	Babol health center 1	CT scan Nowshahr 1	Shohada	Nick Darman
11	Qaemshahr health center 1	CT scan Nor 1	Taleqani	Imam Ali
12	Sari health center 1	CT scan Fereydonkenar 1	17 Shahrivar	Ramsar clinic
13	Neka health center 1	CT scan Mahmoudabad 1	Imam Reza	Tonekabon 1 clinic
14	Sari health center 2	CT scan Amol 1	Shomal	Tonekabon 2 clinic
15	Behshahr health center 1	CT scan Sari 1	Imam Hossein	Iran clinic
16	Galogah health center 1	CT scan Qaemshahr 1	Mehr	Mehregan
17	Fereydonkenar health center 1	CT scan Neka 1	Emam Khomeini Behshahr	Omid
18	Sari health center 3	CT scan Galogah 1	Nowshahr	Boarding Clinic Alborz
19	Behshahr health center 2	CT scan Qaemshahr 2	Imam Sajjad	Hekmat
20	Behshahr health center 4	CT scan Qaemshahr 3	Rajaei	Vali Asr
21	Galogah health center 2	CT scan Qaemshahr 4	Emam Khomeini Fereydonkenar	Velaiat
22	Amol health center 2	CT scan Sari 2	Shafa Sari	Noor
23	Babolsar health center 2	CT scan Sari 3	Bou Ali Sina	Farhangian
24	Nowshahr health center 2	CT scan Sari 4	Khatam Al-Anbia	Darmangah
25	Chalos health center 2	CT scan Neka 2	Shohada	Galogah clinic
26		CT scan Behshahr 2	Samenol Ammeh	Sepid jamegan
27		CT scan Behshahr 3	Ahmadnejad	Tamin Ejtemai
28		CT scan Babolsar 2	Emam Khomeini Nor	Nor clinic
29		CT scan Amol 2	Abbadabad	Imam Reza
30		CT scan Amol 3		Mehr Nowshahr
31		CT scan Nowshahr 2		Shomal
32		CT scan Ramsar 2		

## References

[CR1] Alizadeh M, Paydar MM, Hosseini SM, Makui A (2021). Influenza vaccine supply chain network design during the COVID-19 pandemic considering dynamical demand. Scientia Iranica.

[CR2] Aldrighetti R, Zennaro I, Finco S, Battini D (2019). Healthcare supply chain simulation with disruption considerations: A case study from Northern Italy. Global Journal of Flexible Systems Management.

[CR3] Alinezhad M, Mahdavi I, Hematian M, Tirkolaee EB (2022). A fuzzy multi-objective optimization model for sustainable closed-loop supply chain network design in food industries. Environment, Development and Sustainability.

[CR4] Alizadeh M, Makui A, Paydar MM (2020). Forward and reverse supply chain network design for consumer medical supplies considering biological risk. Computers & Industrial Engineering.

[CR5] Alizadeh M, Sharbafi F, Paydar MM (2020). A bi-objective natural disaster blood supply chain network considering blood transfusion: A case study in Babol. International Journal of Industrial Engineering and Management Science.

[CR6] Armani AM, Hurt DE, Hwang D, McCarthy MC, Scholtz A (2020). Low-tech solutions for the COVID-19 supply chain crisis. Nature Reviews Materials.

[CR9] Birge JR, Louveaux F (2011). Introduction to stochastic programming.

[CR10] Chiaramonti D, Maniatis K (2020). Security of supply, strategic storage and Covid19: Which lessons learnt for renewable and recycled carbon fuels, and their future role in decarbonizing transport?. Applied Energy.

[CR11] Choi TM (2021). Risk analysis in logistics systems: A research agenda during and after the COVID-19 pandemic. Transportation Research Part E: Logistics and Transportation Review.

[CR12] Chowdhury P, Paul SK, Kaisar S, Moktadir MA (2021). COVID-19 pandemic related supply chain studies: A systematic review. Transportation Research Part E: Logistics and Transportation Review..

[CR13] Craighead CW, Ketchen DJ, Darby JL (2020). Pandemics and supply chain management research: Toward a theoretical toolbox. Decision Sciences.

[CR14] Currie CS, Fowler JW, Kotiadis K, Monks T, Onggo BS, Robertson DA, Tako AA (2020). How simulation modelling can help reduce the impact of COVID-19. Journal of Simulation.

[CR15] Dasaklis TK, Pappis CP, Rachaniotis NP (2012). Epidemics control and logistics operations: A review. International Journal of Production Economics.

[CR16] Deaton BJ, Deaton BJ (2020). Food security and Canada’s agricultural system challenged by COVID-19. Canadian Journal of Agricultural Economics/revue Canadienne D'agroeconomie.

[CR17] Dente SMR, Hashimoto S (2020). COVID-19: A pandemic with positive and negative outcomes on resource and waste flows and stocks. Resources, Conservation and Recycling.

[CR19] Gholizadeh H, Jahani H, Abareshi A, Goh M (2021). Sustainable closed-loop supply chain for dairy industry with robust and heuristic optimization. Computers & Industrial Engineering.

[CR20] Govindan K, Mina H, Alavi B (2020). A decision support system for demand management in healthcare supply chains considering the epidemic outbreaks: A case study of coronavirus disease 2019 (COVID-19). Transportation Research Part e: Logistics and Transportation Review.

[CR21] Gray RS (2020). Agriculture, transportation, and the COVID-19 crisis. Canadian Journal of Agricultural Economics/revue Canadienne D'agroeconomie.

[CR22] Homayouni Z, Pishvaee MS, Jahani H, Ivanov D (2021). A robust-heuristic optimization approach to a green supply chain design with consideration of assorted vehicle types and carbon policies under uncertainty. Annals of Operations Research.

[CR24] Ivanov D (2020). Viable supply chain model: integrating agility, resilience and sustainability perspectives: Lessons from and thinking beyond the COVID-19 pandemic. Annals of Operations Research.

[CR25] Ivanov D, Das A (2020). Coronavirus (COVID-19/SARS-CoV-2) and supply chain resilience: A research note. International Journal of Integrated Supply Management.

[CR26] Iyengar KP, Vaishya R, Bahl S, Vaish A (2020). Impact of the coronavirus pandemic on the supply chain in healthcare. British Journal of Healthcare Management.

[CR27] Jabarzadeh Y, Yamchi HR, Kumar V, Ghaffarinasab N (2020). A multi-objective mixed-integer linear model for sustainable fruit closed-loop supply chain network. Management of Environmental Quality: An International Journal..

[CR28] Jahani H, Abbasi B, Hosseinifard Z, Fadaki M, Minas JP (2021). Disruption risk management in service-level agreements. International Journal of Production Research.

[CR29] Jahani H, Abbasi B, Talluri S (2019). Supply chain network redesign: A technical note on optimising financial performance. Decision Sciences.

[CR30] Jahani H, Chaleshtori AE, Khaksar SMS, Aghaie A, Sheu JB (2022). COVID-19 vaccine distribution planning using a congested queuing system: A real case from Australia. Transportation Research Part e: Logistics and Transportation Review.

[CR31] Jaillet, P., Loke, G. G., & Sim, M. (2018). Strategic manpower planning under uncertainty. Available at SSRN 3168168.

[CR32] Kall P, Wallace SW, Kall P (1994). Stochastic programming.

[CR33] Kashanian M, Pishvaee MS, Sahebi H (2020). Sustainable biomass portfolio sourcing plan using multi-stage stochastic programming. Energy.

[CR34] Khalilpourazari, S., & Doulabi, H. H. (2021, August). Using reinforcement learning to forecast the spread of COVID-19 in France. In: 2021 IEEE International Conference on Autonomous Systems (ICAS) (pp. 1–8). IEEE.

[CR35] Khalilpourazari S, Hashemi DH (2022). Designing a hybrid reinforcement learning based algorithm with application in prediction of the COVID-19 pandemic in Quebec. Annals of Operations Research..

[CR36] Khalilpourazari S, Doulabi HH (2021). Robust modelling and prediction of the COVID-19 pandemic in Canada. International Journal of Production Research.

[CR37] Khalilpourazari S, Doulabi HH, Çiftçioğlu AÖ, Weber GW (2021). Gradient-based grey wolf optimizer with Gaussian walk: Application in modelling and prediction of the COVID-19 pandemic. Expert Systems with Applications.

[CR38] Leite H, Lindsay C, Kumar M (2020). COVID-19 outbreak: Implications on healthcare operations. The TQM Journal.

[CR39] Li X, Ghadami A, Drake JM, Rohani P, Epureanu BI (2021). Mathematical model of the feedback between global supply chain disruption and COVID-19 dynamics. Scientific Reports.

[CR40] Lotfi R, Kargar B, Rajabzadeh M, Hesabi F, Özceylan E (2022). Hybrid fuzzy and data-driven robust optimization for resilience and sustainable health care supply chain with vendor-managed inventory approach. International Journal of Fuzzy Systems.

[CR41] Mathur B, Gupta S, Meena ML, Dangayach GS (2018). Healthcare supply chain management: Literature review and some issues. Journal of Advances in Management Research.

[CR42] Nagurney A, Salarpour M, Dong J, Dutta P, Rassias TM, Pardalos PM (2021). Competition for medical supplies under stochastic demand in the Covid-19 pandemic: A Generalized Nash Equilibrium framework. Nonlinear analysis and global optimization.

[CR43] Nagurney A (2021). Optimization of supply chain networks with inclusion of labor: Applications to COVID-19 pandemic disruptions. International Journal of Production Economics.

[CR44] Nikolopoulos K, Punia S, Schäfers A, Tsinopoulos C, Vasilakis C (2021). Forecasting and planning during a pandemic: COVID-19 growth rates, supply chain disruptions, and governmental decisions. European Journal of Operational Research.

[CR45] Nikzamir M, Baradaran V (2020). A healthcare logistic network considering stochastic emission of contamination: Bi-objective model and solution algorithm. Transportation Research Part e: Logistics and Transportation Review.

[CR46] Paul SK, Chowdhury P (2020). A production recovery plan in manufacturing supply chains for a high-demand item during COVID-19. International Journal of Physical Distribution & Logistics Management..

[CR47] Poursoltan L, Seyed-Hosseini SM, Jabbarzadeh A (2021). Green closed-loop supply chain network under the COVID-19 pandemic. Sustainability.

[CR48] Salama MR, McGarvey RG (2021). Resilient supply chain to a global pandemic. International Journal of Production Research.

[CR49] Sazvar Z, Zokaee M, Tavakkoli-Moghaddam R, Salari SAS, Nayeri S (2021). Designing a sustainable closed-loop pharmaceutical supply chain in a competitive market considering demand uncertainty, manufacturer’s brand and waste management. Annals of Operations Research.

[CR50] Scavarda A, Daú GL, Scavarda LF, Korzenowski AL (2019). A proposed healthcare supply chain management framework in the emerging economies with the sustainable lenses: The theory, the practice, and the policy. Resources, Conservation and Recycling.

[CR51] Shabbir MS, Mahmood A, Setiawan R, Nasirin C, Rusdiyanto R, Gazali G, Arshad MA, Khan S, Batool F (2021). Retracted article: Closed-loop supply chain network design with sustainability and resiliency criteria. Environmental Science and Pollution Research.

[CR52] Shahed KS, Azeem A, Ali SM, Moktadir MA (2021). A supply chain disruption risk mitigation model to manage COVID-19 pandemic risk. Environmental Science and Pollution Research.

[CR53] Sousa Jabbour ABL, Jabbour CJC, Hingley M, Vilalta-Perdomo EL, Ramsden G, Twigg D (2020). Sustainability of supply chains in the wake of the coronavirus (COVID-19/SARS-CoV-2) pandemic: Lessons and trends. Modern Supply Chain Research and Applications..

[CR54] Swanson D, Santamaria L (2021). Pandemic supply chain research: A structured literature review and bibliometric network analysis. Logistics.

[CR55] Tirkolaee EB, Goli A, Ghasemi P, Goodarzian F (2022). Designing a sustainable closed-loop supply chain network of face masks during the COVID-19 pandemic: Pareto-based algorithms. Journal of Cleaner Production.

[CR56] Trautrims A, Schleper MC, Cakir MS, Gold S (2020). Survival at the expense of the weakest? Managing modern slavery risks in supply chains during COVID-19. Journal of Risk Research.

[CR58] Zahiri B, Torabi SA, Tavakkoli-Moghaddam R (2017). A novel multi-stage possibilistic stochastic programming approach (with an application in relief distribution planning). Information Sciences.

[CR59] Zeleny M (1976). The attribute-dynamic attitude model (ADAM). Management Science.

